# Absorption Spectra of Flexible Fluorescent Probes by a Combined Computational Approach: Molecular Dynamics Simulations and Time-Dependent Density Functional Theory

**DOI:** 10.1021/acs.jpca.2c04637

**Published:** 2022-11-16

**Authors:** Silvia Di Grande, Ilaria Ciofini, Carlo Adamo, Marco Pagliai, Gianni Cardini

**Affiliations:** † Scuola Superiore Meridionale,Largo San Marcellino 10, I-80138Napoli, Italy; ‡ Scuola Normale Superiore, Piazza dei Cavalieri 7, I-56126Pisa, Italy; ¶ Department of Chemical Sciences, University of Napoli Federico II, Complesso Universitario di M.S. Angelo, via Cintia 21, I-80126Napoli, Italy; § PSL University, Chimie ParisTech-PSL, CNRS, Institute of Chemistry for Health and Life Sciences (iCLeHS UMR8060), F-75005Paris, France; ⊥ Institut Universitaire de France, 103 Boulevard Saint Michel, F-75005Paris, France; # Dipartimento di Chimica “Ugo Schiff”, Università degli Studi di Firenze, Via della Lastruccia 3, Sesto FiorentinoI-50019, Italy

## Abstract

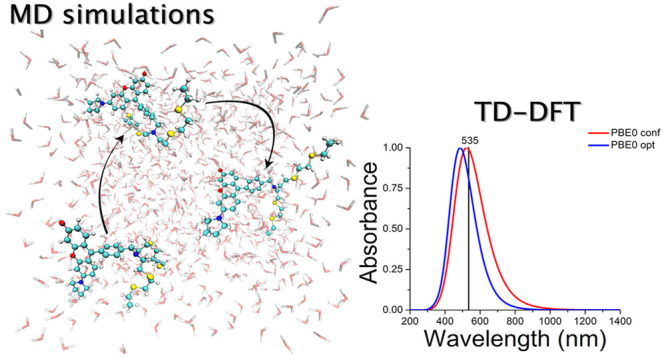

A detailed understanding and interpretation of absorption spectra of molecular systems, especially in condensed phases, requires computational models that allow their structural and electronic features to be connected to the observed macroscopic spectra. This work is focused on modeling the electronic absorption spectrum of a fluorescent probe, namely, the 9-(4-((bis(2-((2-(ethylthio)ethyl)thio)ethyl)amino)methyl)phenyl)-6-(pyrrolidin-1-yl)-3*H*-xanthen-3-one molecule, depicted by a combined classical-quantum chemical approach. Particularly, first classical molecular dynamics (MD) has been used to explore the configurational space, and next, the absorption spectrum has been reconstructed by averaging the results of time-dependent density functional theory (TD-DFT) calculations performed on equispaced molecular conformations extracted from MD to properly sample the configurational space explored at finite temperature. To verify the effect of molecular conformation on the spectral profile, the generated electronic absorption spectra were compared with those obtained considering a single structure corresponding to the optimized one, an approach also referred to as static. This comparison allows one to highlight a sizable though small shift between the maxima of the corresponding reconstructed absorption spectra, highlighting the importance of conformational sampling in the case of this rather flexible molecule. Four different exchange and correlation functionals (PBE, BLYP, PBE0, B3LYP) were considered to compute vertical transition via TD-DFT calculations. From the results obtained in gas and in condensed, here solution, phases, it appears that the magnitude of the shift is actually more affected by the phase in which the system is found than by the functional used. This fact underlines the central importance of conformational mobility, that is flexibility, of this molecule. From a more quantitative point of view, a comparison with available experimental data shows that hybrid functionals, such as PBE0 and B3LYP, enable one to faithfully reproduce the observed absorption maxima.

## 1 Introduction

In the past decades, the prediction of optical properties of molecular compounds has become an increasingly active and captivating research field. Being able to predict and simulate the optical properties of dyes or fluorescent probes, such as maximum wavelengths or the profiles of absorption and/or emission spectra, paves the route for their understanding and their *in silico* assisted design.^1−3^ The possibility of controlling the spectroscopic properties of a molecule, by modifying its skeleton, finds applications in many fields from medical to technological and industrial ones.^4^ For this reason, over the years, large efforts have been spent to develop *ab initio* and nonparametrized methods enabling one to accurately probe the excited electronic states of dyes and fluorescent molecules. These approaches can be divided into two categories: wave function-based methods and density-based methods.^5^ The former, despite their accuracy, are limited by their computational burden, while methods rooted on time-dependent density functional theory (TD-DFT) though known to have intrinsic deficiencies still represent a good compromise between accuracy and computational cost, consequently representing the method of choice for the study of large systems in many cases.^6,7^

Many studies using TD-DFT have been carried out to reproduce the spectroscopic properties of fluorescent dye molecules.^8−23^ Of course, the accuracy of the results is highly dependent on the choice of the exchange and correlation functional. Therefore, investigations were performed to determine the exchange and correlation functional that best reproduces the spectroscopic properties of fluorescent dye molecules.^24^ From these studies, it was shown that, for dyes comprising excited states of limited charge transfer character, hybrid functionals^25^ and, in particular, those casting a low fraction of exact exchange such as B3LYP^26^ or PBE0^27^ usually provide the best accuracy.^4,5,12,15,20,28^

Nonetheless, a large part of the benchmarks and of the above-mentioned applications concerns the prediction of photophysical properties of rather rigid dyes, which nonetheless represent an important fraction of the molecules of relevance for industrial application.^29,30^ The situation is indeed more delicate in the case of flexible molecules. In this case, the accuracy obtained can be significantly reduced since there is an additional complexity represented by the presence of several energetically accessible minima in the potential energy surfaces (PESs) of both the ground state (GS) and the excited states. Therefore, a simple procedure of minimization, providing only one of the possible accessible structures, may fail to reproduce the experimentally observed spectrum that results from the weighted average of all spectra associated with all the thermally populated molecular conformations. Hence, to reproduce the experimental data, it is no more sufficient to compute the absorption spectrum associated with a single molecular conformation in the structural minimum, but it is necessary to compare an average of the spectrum corresponding to the properly thermally weighted structures. Two ingredients shall thus be mixed: (i) a method enabling one to generate an *ensemble* of statistically relevant conformations and (ii) a method enabling one to compute, in a cost efficient way, the desired photophysical property on such structures.

Two main strategies can be applied to accomplish the first task: a Monte Carlo (MC) exploration^31^ or molecular dynamics (MD) simulations.^32−35^

Here, we will adopt the second strategy, and the spectrum will be reconstructed as a weighted sum of the spectra computed at the TD-DFT level on equispaced molecular conformations extracted from MD simulations. Particularly, we will analyze the effect of different computational or physical parameters (such as the phase in which the system is found) on the spectral profile.

Due to the dimension and the shape of the molecule subject of this work, we did not try to model the full absorption profile that would require more time-consuming calculations,^36^ such as taking into account vibronic coupling, or perform *ab initio* MD simulations to sample the phase space, or consider explicitly the first solvation shell in the TD-DFT calculations.

**Figure 1:**
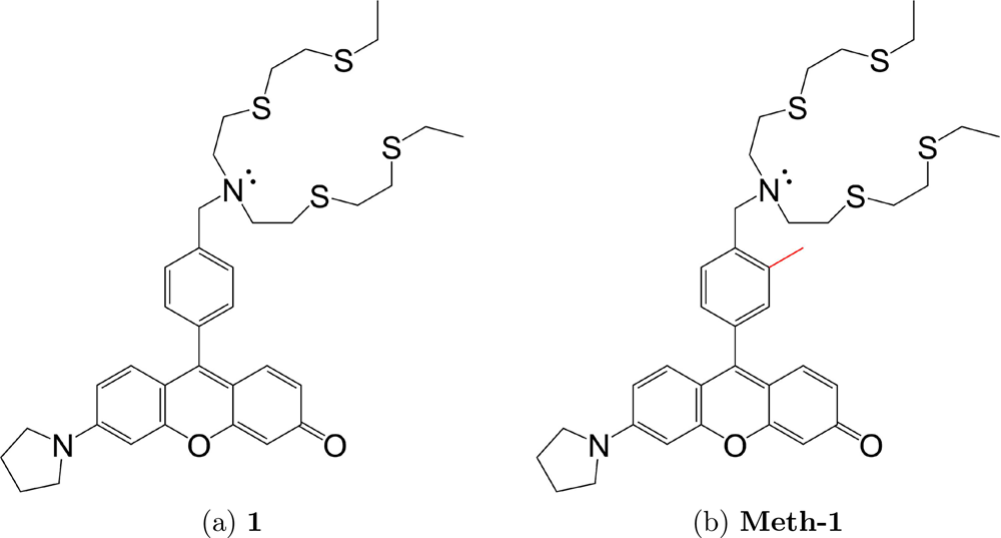
(a, b) Structure of the systems investigated in this work. In (b), the common structure of the two molecules is represented in black and the different group in red.

Two molecules will be studied, namely, the 9-(4-((bis(2-((2-(ethylthio)ethyl)thio)ethyl)amino)methyl)phenyl)-6-(pyrrolidin-1-yl)-3*H*-xanthen-3-one model system (**1**, Figure 1) as well as its experimentally characterized methyl substituted derivate (**Meth-1**, Figure 1). These molecules, closely related to fluorescein and rhodamine, are part of a family of fluorescent probes used for copper recognition, recently developed to study the role of this metal in the physiological environment.^37,38^ Since the experimental absorption spectra in solution are available for the **Meth-1** molecule,^38^ these data will be used to evaluate the overall accuracy of the computational procedure.

Besides their interest related to possible applications, these molecules were chosen for their expected flexibility, which will be quantitatively evaluated through the analysis of the evolution of selected structural parameters along the MD simulations. Due to the presence of a rather flat PES at the ground state, these molecules represent a perfect playground to test the robustness of the computational procedure for simulating the electron absorption spectra. We will thus first highlight the close behavior of **1** and **Meth-1** based on structural and photophysical properties obtained for the optimized compounds and then proceed, in the case of the model compound **1**, to a deeper comparison between the spectra obtained from minimal energy (static approach, hereafter labeled as opt) or PES sampling, hereafter labeled as conf.

## 2 Computational Procedure

As already mentioned in the Introduction, we will make use of either a static or sampling procedure to identify relevant conformers to be used to compute the electronic absorption spectra. In the latter case, the computational procedure consists in two steps: (i) sampling of the ground state PES using classical molecular dynamics followed by (ii) reconstruction of the spectra obtained from an average of the TD-DFT spectra computed on statistically representative structures extracted from the MD trajectories. This procedure has been applied to simulate the spectra both in the gas phase and in a water solution in the case of molecule **1**. Below, the computational details associated with each of these steps are detailed.

### 2.1 Ground State Sampling via Classical Molecular Dynamics Simulations

For MD simulations, both in the gas phase and in solution, the primaDORAC web interface^39^ was used to produce the topology and parameters files. The force field (FF) is generated according to the GAFF2 protocol, and all the simulations were performed with the GROMACS package,^40^ version 2019.6. The electrostatic part has been treated through the Smooth Particle-Mesh Ewald (SPME) algorithm.^41^ Preliminarily, the accuracy of the semiempirical FF in reproducing the molecular structures of the studied systems with respect to the DFT calculations has been verified, as reported in Section S1 of the Supporting Information. The comparison of the results allows us to adopt with confidence the FF in MD simulations and subsequent TD-DFT calculations.

1250 sufficiently uncorrelated configurations, sampled every 4 ps, were obtained from each MD simulation and analyzed with the VMD program^42^ to monitor the evolution of structural parameters such as dihedral angles, as reported in Section S2 of the Supporting Information.

To simulate the behavior of the molecules in water solution, 2048 water molecules have been added using a 40 Å side cubic cell. Since the TIP3P water model does not accurately describe the hydrogen bond, a more accurate model was chosen, the so-called TIP3P-FB.^43−46^

The MD simulation consists of an initial equilibration part followed by the accumulation run in the NPT *ensemble*.

Particularly, the first part consists in a first energy minimization (50,000 steps), performed keeping the water bonds constrained, followed by a second one (500,000 steps) with flexible water bonds. Both minimizations were performed with a steepest descent algorithm. After these two energy minimizations, two equilibration runs were performed, the first in the NVT *ensemble* and the second in the NPT *ensemble*. The equations of motion have been integrated using a time step of 2 fs; the time length of the two thermalization runs is 100 ps and 2 ns, respectively. For the NVT simulation, a Nose-Hoover chain algorithm has been adopted with a 1 ps time constant and a 298.15 K reference temperature. The 1 ps time constant controls the period of the temperature fluctuations at equilibrium. A Parrinello–Rahman extended Lagrangian^47,48^ was employed for the NPT equilibration with a time constant of 2 ps with the fixed cubic cell constraint. The reference pressure for coupling is 1 bar, and the compressibility is 4.46 × 10^–5^ bar^–1^. The last part of the simulation is represented by an accumulation run of 5 ns that allows a good sampling of the accessible phase space.

In the gas-phase molecular dynamics simulations, a single molecule was isolated in a 50 Å side cubic box. In this case, the second thermalization run (i.e., the NPT equilibration) was not carried out. When the simulation cell is enlarged from 40 to 50 Å, the system can indeed be considered isolated, therefore requiring only one NVT equilibration.

### 2.2 Calculation of the Electronic Absorption Spectrum Using TD-DFT

To reconstruct the absorption spectrum, 126 configurations were extracted from the 1250 obtained from the classical molecular dynamics simulations. The sampling was done by extracting one configuration for every 10, starting from the first one saved from the MD runs. Vertical TD-DFT calculations on the extracted configurations were performed with the Gaussian 16 software package^49^ with four different functionals, namely, B3LYP,^50^ BLYP,^51,52^ PBE0,^27^ and PBE,^53^ using the 6-31+G(d, p) basis set.^54^ Solvent effects, contrary to MD simulations, where the solvent is considered explicitly, have been included implicitly using a continuum polarizable model, CPCM.^55,56^ This allows us to take into account the effect of the dielectric constant of the solvent, which is the first order effect since the molecular conformation has been determined by the explicit interaction with the solvent, saving huge amounts of computer resources with respect to explicitly considering the molecules of a few solvation shells. The effects of the different levels of theory in producing the molecular conformation and the one used in the TD-DFT calculations are not considered in the present work, since they are a second order effect on the electronic transition with respect to the use of uncorrelated molecular configurations.

From these calculations, the energy and oscillator strength associated with a number of excited states, five or ten, allowing one to cover the visible spectra, have been derived. Particularly, for the B3LYP and PBE0 functionals, both in the gas phase and in solution, five excited states were computed with the only exception of one B3LYP gas-phase configuration (number 56 referring to the 126 extracted configurations) for which the first ten excited states were computed. As it concerns BLYP and PBE, ten excited states were considered for the gas phase and five in solution. Starting from these data, the resulting absorption spectrum (*A*[*E*]) was simulated by associating a normalized Gaussian function (*g*) centered on the vertical excitation energies, (Δ*E*_*IL*_(**R**_*k*_)), between the ground electronic state (*I*) and excited state (*L*) with a full width at half-maximum (δ) of 0.2 eV and multiplied by the corresponding oscillator strengths (*f*_*IL*_(**R**_*k*_)), as reported in eq 1:
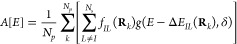
1where *N*_*s*_ is the number of excited states considered and *N*_*p*_ is the number of sampled points.^31^

Finally, TD-DFT calculations were performed with the four different functionals chosen both in the gas phase and in solution also for the corresponding, DFT optimized, molecular structure. Ground state structural optimizations were performed using the same basis set and functionals used to perform TD-DFT calculations with the Gaussian 16 software package.^49^ These additional calculations allow a comparison between the absorption spectrum of the molecule in its energy minimum with that stemming from a statistically relevant population of possible conformations obtained via MD simulations, as previously explained.

## 3 Results and Discussion

### 3.1 Simulation of the Absorption Spectra Using a Static Approach

**Figure 2:**
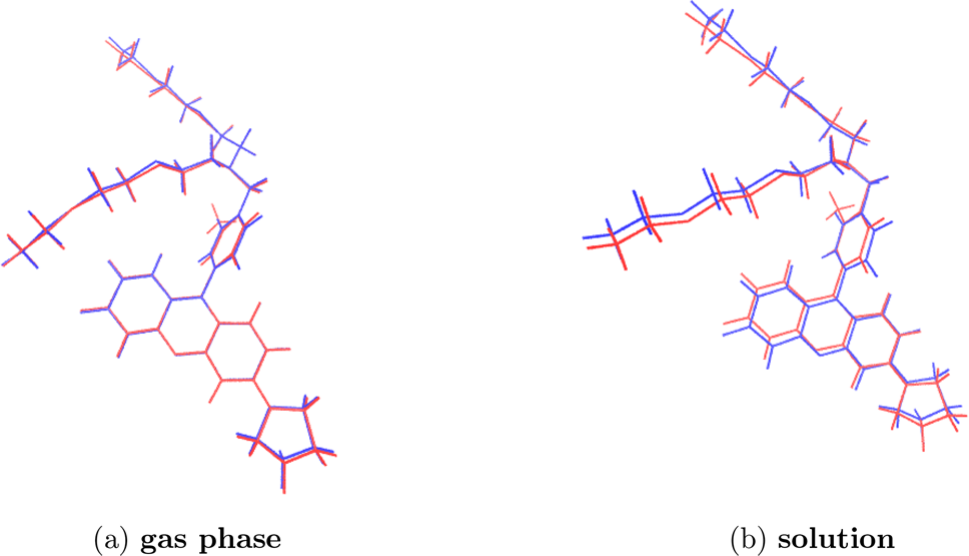
Comparison between the optimized structures of **1** (blue) and **Meth-1** (red) computed at the PBE0 level.

First, we will briefly comment on the electronic transitions mainly contributing to the spectra focusing on the TD-DFT results obtained using optimized structures. Since all functionals considered, though providing a slightly different maximum of absorbance (see below the discussion and results reported in Table 1), display completely equivalent results in term of the nature of the electronic transitions involved, in the main text, we will discuss in detail only the results obtained with the PBE0 functional, with all corresponding data for PBE, BLYP, and B3LYP being reported in the Supporting Information (Section S3). First of all, it is worth mentioning that from a structural point of view the introduction of a methyl substituent on the skeleton of molecule **1** does not induce any relevant change on the optimized structure both in the gas phase and in water solution as evident from the data reported in Figure 2. This conclusion can be extended to a different extent to all the other functionals considered not showing any major difference between the structure of the **Meth-1** and **1** molecules, if one excludes a different arrangement of the thioethyl chain substituents on the amino group, which indeed, as we will see later, are not involved in the electronic transitions of interest.

**Table 1:** Computed λ_max_ (in nm) for Molecules **1** and **Meth-1** at Various Levels of Theory, Considering Either a Single Spectra Computed on the DFT Optimized GS Structure (opt) or an Average Extracted from MD Conformations (conf), Together with Their Shift (Δ*E*, in eV)^a^

	B3LYP			BLYP			PBE0			PBE			
	conf	opt	Δ*E*	conf	opt	Δ*E*	conf	opt	Δ*E*	conf	opt	Δ*E*	exp.^38^
gas phase **1**	471	426	0.275	523	477	0.228	458	418	0.261	525	478	0.229	
gas phase **Meth-1**		425			478			416			480		
solution **1**	535	500	0.164	590	561	0.106	523	486	0.179	592	560	0.122	
solution Meth-1		491			558			476			554		535

a For molecule **Meth-1**, only data corresponding to a static approach (opt) have been computed (see the text for an explanation and details). The experimental absorption λ_max_ of **Meth-1** is also reported. The experimental spectrum taken from ref (38) was recorded on an aqueous solution, prepared with Milli-Q water, in which a buffer was added to maintain a neutral pH. The absorption spectrum was recorded using a Varian Cary 50 spectrophotometer, and the sample for the absorption measurement was contained in a 1 cm × 1 cm quartz cuvette.^38^

**Figure 3:**
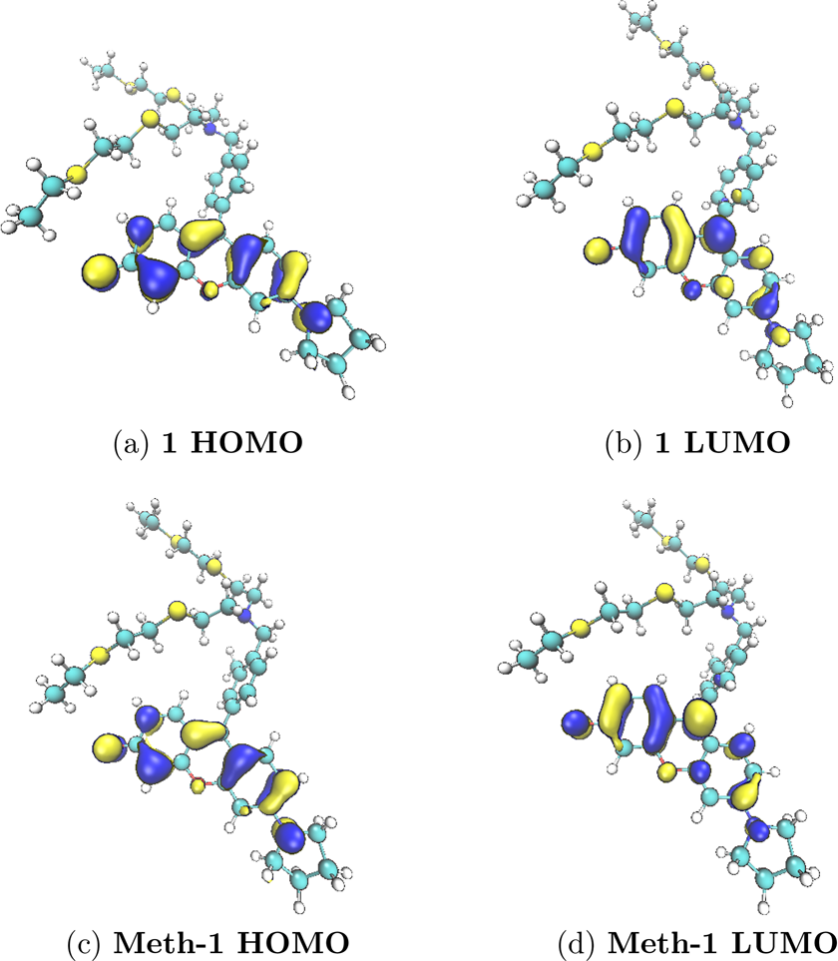
Isovalue representation of the molecular orbitals (computed at the PBE0 level in the gas phase) mainly involved in the bright electronic transition responsible for the absorption in the visible range.

The analysis of the vertical electronic transitions occurring in the visible range shows, independently of the functional and the phase considered, that a single bright electronic excitation is responsible for the absorption band experimentally observed. Both in the case of **Meth-1** and **1**, this transition is of π–π* character. Both these orbitals are actually well localized on the xanthene core of the molecule with a very small contribution of the pyrrolidine substituent, as reported in Figure 3 in the case of gas-phase calculations. A completely similar picture can be drawn in the case of calculations performed in solution and independently on the functional as substantiated by the raw data reported in Section S3 of the Supporting Information.

Already from these data, one can thus expect hybrid functionals, such as PBE0 and B3LYP, to accurately describe the electronic spectra of these molecules characterized by a transition of limited charge transfer character. A detailed listing of all vertical electronic transition energies and character, computed in gas phase and solution considering the optimized structures, is provided in the Supporting Information together with corresponding convoluted spectra.

From the convoluted spectra obtained with all functionals, it is possible to extract the resulting maxima of absorbance (λ_max_) in the gas phase and in solution predicted by the static approach, which are reported in Table 1 under the label opt.

A comparison between data computed on optimized systems for both **1** and **Meth-1** shows that the difference in absorption maxima both in the gas phase and in solution are practically negligible with a maximum shift of 10 nm in the case of PBE0 in the water solution. Therefore, in the following, the more expensive simulation of the spectra stemming from the structures generated via MD simulations will only be performed in the case of the model compound **1**, even if experimental data are available for compound **Meth-1**. Of note, there is a quite important dispersion in the computed λ_max_ that spans from 478 to 477 nm (for PBE and BLYP) to 418 to 426 nm (for PBE0 and B3LYP) in the gas phase and from 560 to 561 nm (for PBE and BLYP) to 486 to 500 nm (for PBE0 and B3LYP). Indeed, GGA functionals are found to predict red-shifted values with respect to hybrids in full agreement with several previous reports.^4,57,58^

### 3.2 Simulation of the Absorption Spectra Using an MD Exploration of the PES

**Figure 4:**
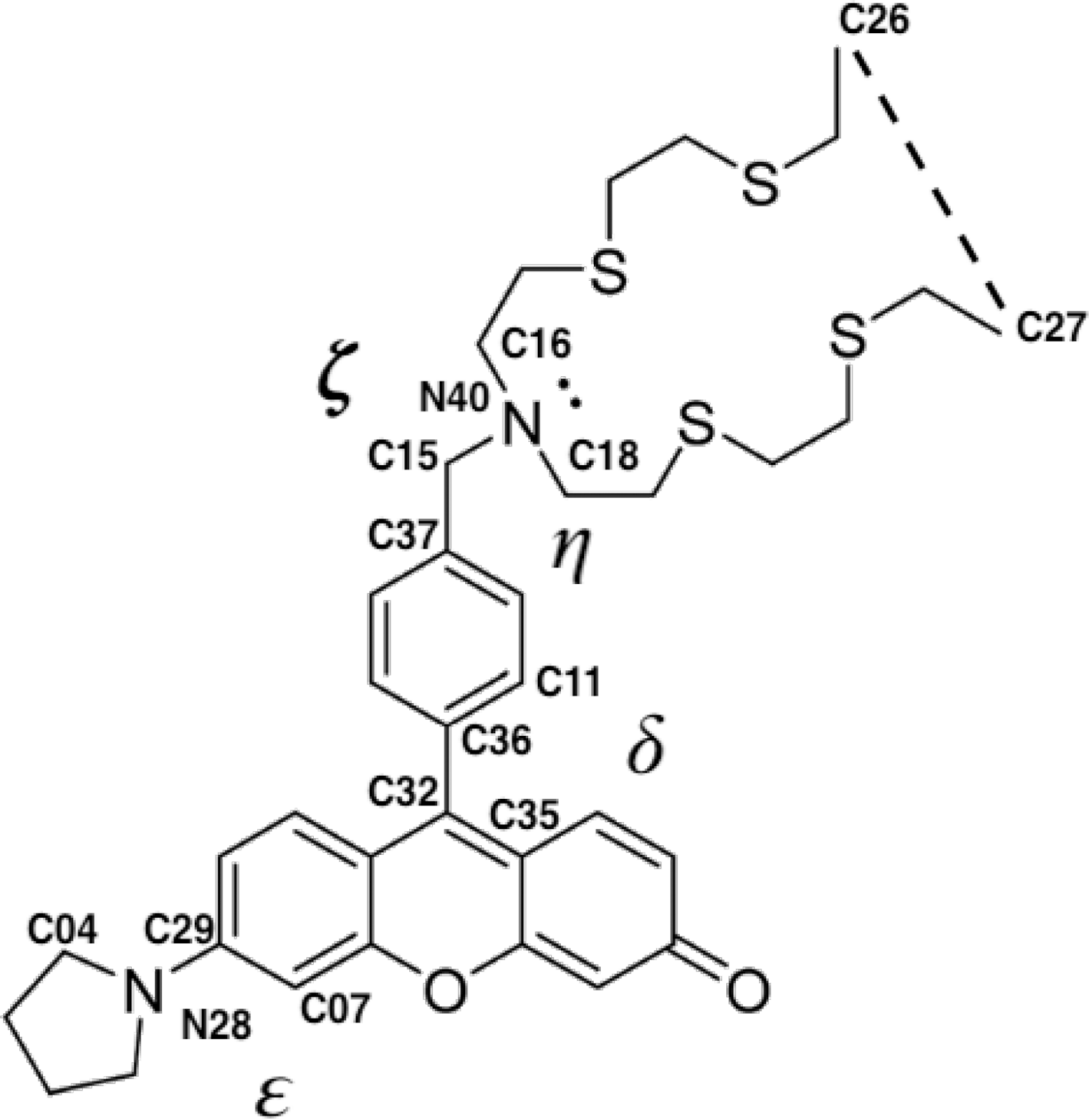
Dihedral angles δ (C11–C36–C32–C35), ε (C04–N28–C29–C07), ζ (C16–N40–C15–C37), and η (C18–N40–C15–C37) monitored along the MD trajectories together with the interchain distance (C27–C26).

As detailed in the Computational Procedure, first of all, an exploration of the PES has been performed using classical MD. From these simulations, it is possible to monitor the distribution of geometrical parameters and define the most populated conformations at a given temperature. Here, due to the very rigid nature of the xanthene core, it is expected that the most relevant parameters allowing one to monitor the flexibility of the molecules will be represented by the torsional angles ruling the relative orientation of the substituents with respect to the central core. These torsional angles will also rule the conjugation of the peripheral substituents with the xanthene core and are indeed expected to be those most affecting the predicted spectra, since they can induce a modification of the π system extension and delocalization. Furthermore, since, in applications, the thio substituted chains are expected to coordinate copper, another parameter that may be of interest to monitor although not necessarily impacting the spectral properties is the distance between the two chains. For this reason, the four dihedral angles (δ, ε, η, ζ) and the distance between the terminal carbon atoms of the alkyl chains, depicted in Figure 4 (in the case of **1**), have been monitored.

**Figure 5:**
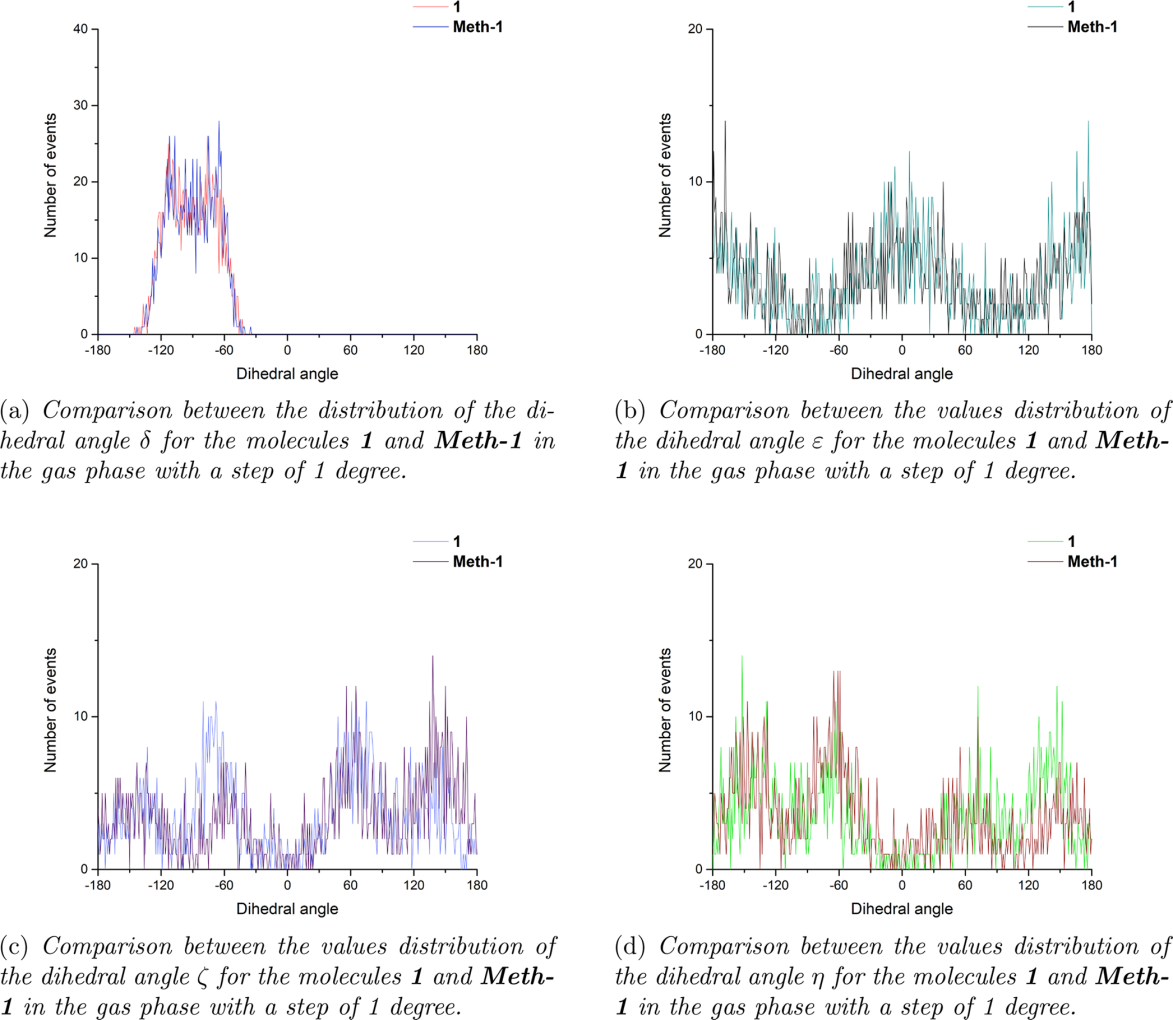
Comparison between distributions of the dihedral angles computed in the gas phase for the molecules **1** and **Meth-1**.

First of all, independently of the phase, gas or solution, the distributions computed for these four dihedral angles for **1** and **Meth-1** all look very similar, as it can easily inferred by the data reported in Figure 5 for the gas phase and in the Supporting Information (Section S2) for the solution. These data confirm the close analogy of the two molecules and fully justify the choice of performing the analysis of the spectra properties derived from the MD simulations only in the case of the model compound **1**. Analysis of the values of the dihedral angle corresponding to maxima in each of the distributions (reported in Table 2) shows that actually the solvent only slightly shifts the maximal values but, for instance, in the case of δ and ε ruling the coupling with the central xanthene core does not significantly affect their distribution. Comparing these values with those obtained from a static approach at the PBE0 level (reported in Table 2 under the label opt) allows one to show how classical MD is able to provide the DFT minimal energy structure among those of the most populated conformers, both in the gas phase and in solution. Indeed, optimized DFT structures for both **1** and **Meth-1** are characterized by dihedrals in line with those corresponding to the maxima of the distributions extracted from MD simulations. Nonetheless, and more interestingly, the distributions computed from MD simulations clearly show that structural fluctuations around these most probable configurations are indeed possible at room temperature. These latter will directly influence the spectra obtained from an MD-based sampling and shift them with respect to those computed using a static approach.

**Table 2:** Values of the Dihedral Angles (in Degrees) Corresponding to a Maximum on the Distributions Obtained from MD Simulations in the Gas Phase and Solution for Molecules **1** and **Meth-1** Together with Those (Labeled as opt) Corresponding to the Optimized Structures Obtained Using the PBE0 Functional

	δ	ε	η	ζ
gas phase **1**	–113 ± 1,–76 ± 1	177 ± 1	–152 ± 1	65 ± 1
gas phase **Meth-1**	−109.5 ± 3.5,–65 ± 1	–168 ± 1	–62 ± 4	138 ± 1
gas phase **1** opt	–68	–175	63	–166
gas phase Meth-1 opt	–72	–175	60	–169
solution **1**	–115 ± 1,–64 ± 1	–7 ± 5	–55 ± 1	–80 ± 1
solution Meth-1	–119 ± 1,–67 ± 9	179 ± 1	102 ± 11	–116 ± 1
solution **1** opt	–66	–176	64	–165
solution Meth-1 opt	–66	–176	63	–166

**Figure 6:**
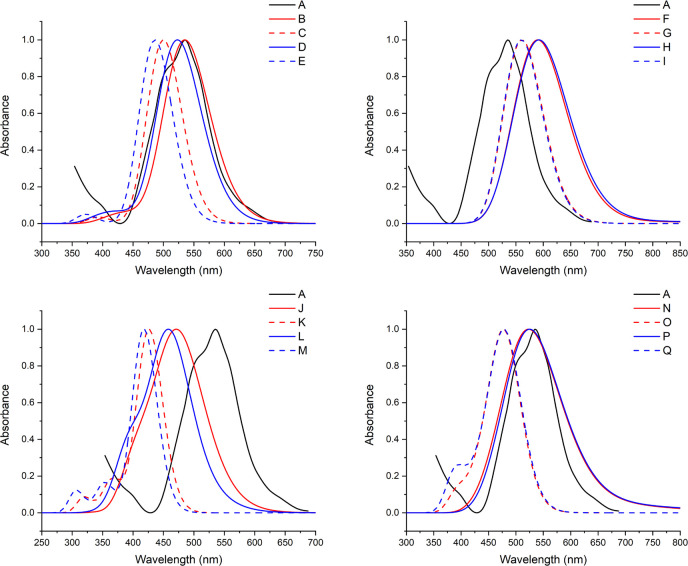
Normalized absorption spectra of **1** computed at the TD-DFT level using different functionals on the optimized structure (opt) or on MD conformers (conf) in solution (upper panels) and in the gas phase (bottom panels) with the experimental absorption spectrum.^38^ (A) Experimental spectrum, (B) B3LYP/6-31+G(d, p)/CPCM/conf, (C) B3LYP/6-31+G(d, p)/CPCM/opt, (D) PBE0/6-31+G(d, p)/CPCM/conf, (E) PBE0/6-31+G(d, p)/CPCM/opt, (F) BLYP/6-31+G(d, p)/CPCM/conf, (G) BLYP/6-31+G(d, p)/CPCM/opt, (H) PBE/6-31+G(d, p)/CPCM/conf, (I) PBE/6-31+G(d, p)/CPCM/opt, (J) B3LYP/6-31+G(d, p)/conf, (K) B3LYP/6-31+G(d, p)/opt, (L) PBE0/6-31+G(d, p)/conf, (M) PBE0/6-31+G(d, p)/opt, (N) BLYP/6-31+G(d, p)/conf, (O) BLYP/6-31+G(d, p)/opt, (P) PBE/6-31+G(d, p)/conf, and (Q) PBE/6-31+G(d, p)/opt.

In Figure 6 are reported the normalized absorption spectra computed either in the gas phase or in solution using a MD sampling (conf) and the optimized structure (opt) using the different exchange correlation functionals. The value of the maximum of the experimental absorption peak^38^ is also reported for comparison, while maxima derived from the computed spectra are reported in Table 1.

First, we will start from the analysis of the effect of the functional on the computed λ_max_. Independently of the sampling (opt or conf) and of the phase (gas or solution), results obtained with GGA functionals (PBE and BLYP) using the same approach (that opt or conf in gas phase or in solution) are very close (with a maximal shift of only 2 nm) and systematically red-shifted with respect to the values computed using the corresponding hybrid functionals (i.e., PBE0 and B3LYP). These latter also show extremely similar results although the differences between PBE0 and B3LYP values are slightly larger (on the order of 12–13 nm). Inclusion of the solvent induces a marked red-shift of the predicted λ_max_ with respect to gas-phase results, independently of the functional and the sampling mode (opt or conf). This shift is slightly larger when the optimized structures are considered, ranging from 84 to 82 nm (computed at the BLYP and PBE levels) to 74 to 68 nm (computed at B3LYP and PBE0, respectively) and practically constant at GGA (BLYP or PBE, 67 nm) and hybrid (BL3YP and PBE0, 64–65 nm) levels, when averaging on MD structures is performed (conf approach). More relevantly, from a methodological point of view, both in the gas phase and in solution, there is a constant red-shift (both for GGA and hybrid functionals) between the λ_max_ computed on a single optimized structure (opt values) and the corresponding values stemming from an MD sampling. This shift, reported in Table 1 under the column Δ*E*, is actually on the order of 0.2 eV (from 0.275 eV for B3LYP to 0.228 eV for BLYP) in the gas phase, and it is reduced to 0.1 eV (from 0.179 for PBE0 to 0.106 eV for BLYP) in solution.

**Figure 7:**
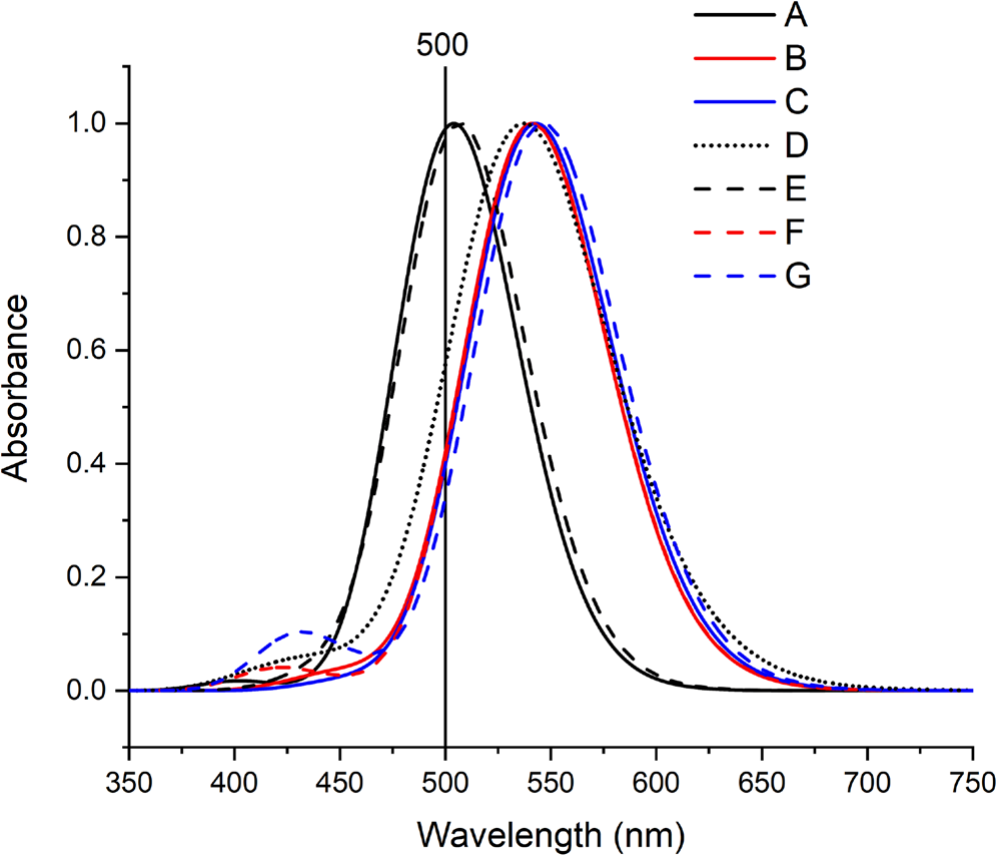
Comparison between the normalized absorption spectra computed in aqueous solution with the B3LYP functional for the molecules **1** and **Meth-1** in different conformations. (A) **1** δ = −88 ± 1°, (B) δ = −115 ± 1°, (C) δ = −64 ± 1°, (D) spectra of **1** resulting from a full conformational sampling, (E) **Meth-1** δ = −88 ± 1°, (F) **Meth-1** δ = −115 ± 1°, and (G) **Meth-1** δ = −115 ± 1°. The value δ = −88 ± 1° corresponds to the minimum (see the text for explanation) together with the maxima computed using the optimized structure in solution (500 nm).

This shift is due to the population, during the molecular dynamics simulations, of conformations that do not correspond to the energy minimum. The smaller shift between opt and conf approaches observed in the case of calculations performed in solution highlights that the conformational mobility of the molecule in solution is actually reduced. Further analysis allows one to also identify the δ dihedral angle as the most relevant in inducing a shift in the absorption band. To support this assertion, in Figure 7 is reported the comparison between the normalized absorption spectra, computed in an aqueous solution using the B3LYP functional, corresponding to three different conformations characterized by different δ values: one (−88°) corresponding to the minimum and the two others, to the two maxima of the bimodal distribution of the angle.

Finally, it should be noted that inclusion of conformational averaging allows one to obtain in the case of hybrid functionals an excellent agreement with the observed λ_max_ with values of 535 and 523 nm for B3LYP and PBE0, respectively, with respect to a value of 535 nm experimentally measured for **Meth-1**. Besides confirming that the approach proposed here is suitable for the investigation of flexible systems, our data also show that the conclusion that one could derive on the basis of a static approach for which GGA functionals provide a better agreement with the experimental data with respect to hybrids is indeed only due to an error compensation between the red-shift induced by the use of GGA and the absence of conformational averaging.

## 4 Conclusions

This work, performed on a simple yet rather flexible molecular fluorophore, made it possible to evaluate the importance of correct sampling of the configurational space when aiming to accurately determine both structural and dynamic features of relevance for the calculation of photophysical properties at the finite, here room, temperature. The applied methodology based on the combination of MD simulations and TD-DFT calculations has allowed us to accurately reproduce the absorption spectra of the studied fluorescent probes in the condensed phase, here, a water solution. A comparison of the results obtained using the multistep protocol using four different exchange and correlation functionals (BLYP, PBE, B3LYP, PBE0) has shown that hybrid functionals (B3LYP and PBE0) provide absorption spectra in excellent agreement with the experiment. Moreover, the comparison of the results obtained using a static approach (making use of a single optimized geometry) with the approach including an average of the conformations, obtained by molecular dynamics simulations, has shown that the torsional mobility affects the spectral absorption profile and that a correct sampling of the configurational space is, therefore, required to successfully reproduce the spectroscopic properties. When the same methodology both for the gas phase and for the aqueous solution was applied, it was possible to highlight a sizable effect of the solvent in reducing the molecular flexibility. Furthermore, in this work, we have shown the importance of a correct sampling of the conformational phase space to reproduce the experimental spectra and how the shift can be related to an internal degree of freedom from the analysis of the simulated data. This suggests that, once the degrees of freedom that give the main contribution to the experimental spectra are identified, these could be used to compare different functionals and limit the TD-DFT calculations to few conformational configurations with a good savings of computational resources. This last point allows, from the point of view of the applications, the *in silico* identification of the most relevant structural parameters that govern the observed spectra, and it is clearly of central interest for the computational design of new systems.

## Supporting Information Available

Section S1: Structural analysis of the optimized molecule **1**; Section S2: Structural analysis derived from molecular dynamics simulations of **1** and **Meth-1**; Section S3: DFT and TD-DFT results obtained with the four different exchange and correlation functionals (BLYP, PBE, B3LYP, PBE0) both in the gas phase and in solution (PDF)
