# Supplementary material for: Absorption Spectra of Flexible Fluorescent Probes by a Combined Computational Approach: Molecular Dynamics Simulations and Time-Dependent Density Functional Theory
Source: J Phys Chem A. 2022 Nov 16;126(47):8809–17. doi: 10.1021/acs.jpca.2c04637 (PMC9720718; doi:10.1021/acs.jpca.2c04637)
Supplement: Supplementary file 1 — jp2c04637_si_001.pdf [file jp2c04637_si_001.pdf]

Supporting Information
Absorption Spectra of Flexible Fluorescent Probes by a Combined Computational Approach: Molecular Dynamics Simulations and TD-DFT

Silvia Di Grande,[*,†,‡,¶] Ilaria Ciofini,[*,§] Carlo Adamo,[*,§,∥] Marco Pagliai,[*,⊥] and Gianni Cardini[*,⊥]

†*Scuola Superiore Meridionale, Largo San Marcellino 10, I-80138 Napoli, Italy*

‡*Scuola Normale Superiore, Piazza dei Cavalieri 7, I-56126 Pisa, Italy*

¶*Department of Chemical Sciences, University of Napoli Federico II, Complesso Universitario di M.S. Angelo, via Cintia 21, I-80126 Napoli, Italy*

§*PSL University, Chimie ParisTech-PSL, CNRS, Institute of Chemistry for Health and Life Sciences, F-75005 Paris, France*

∥*Institut Universitaire de France, 103 Boulevard Saint Michel, F-75005 Paris, France*

⊥*Dipartimento di Chimica "Ugo Schiff", Universitá degli Studi di Firenze, Via della Lastruccia 3, Sesto Fiorentino I-50019 Italy*

E-mail: silvia.digrande@sns.it; ilaria.ciofini@chimieparistech.psl.eu; carlo.adamo@chimieparistech.psl.eu; marco.pagliai@unifi.it; gianni.cardini@unifi.it

Contents

S1 Structural analysis of the optimized molecule 1 **S2**

S2 Structural analysis derived from MD simulations of the molecules 1 and Meth-1 in the gas phase and in solution **S15**

S3 DFT and TD-DFT results: molecular orbitals analysis, detailed transition energies and optimized structures **S21**

S3.1 PBE0 results in the gas phase S21

S3.2 PBE0 results in solution S24

S3.3 PBE results in the gas phase S27

S3.4 PBE results in solution S31

S3.5 B3LYP results in the gas phase S34

S3.6 B3LYP results in solution S37

S3.7 BLYP results in the gas phase S40

S3.8 BLYP results in solution S44

S1 Structural analysis of the optimized molecule 1

The structural parameters of the optimized isolated molecule with the four exchange and correlation functionals were compared against the ones obtained with the chosen force field (FF) to evaluate its goodness. Tables S1 and S2 show the comparison of structural parameters, lengths and bond angles, respectively, referring to Figure S1 for atoms numeration.

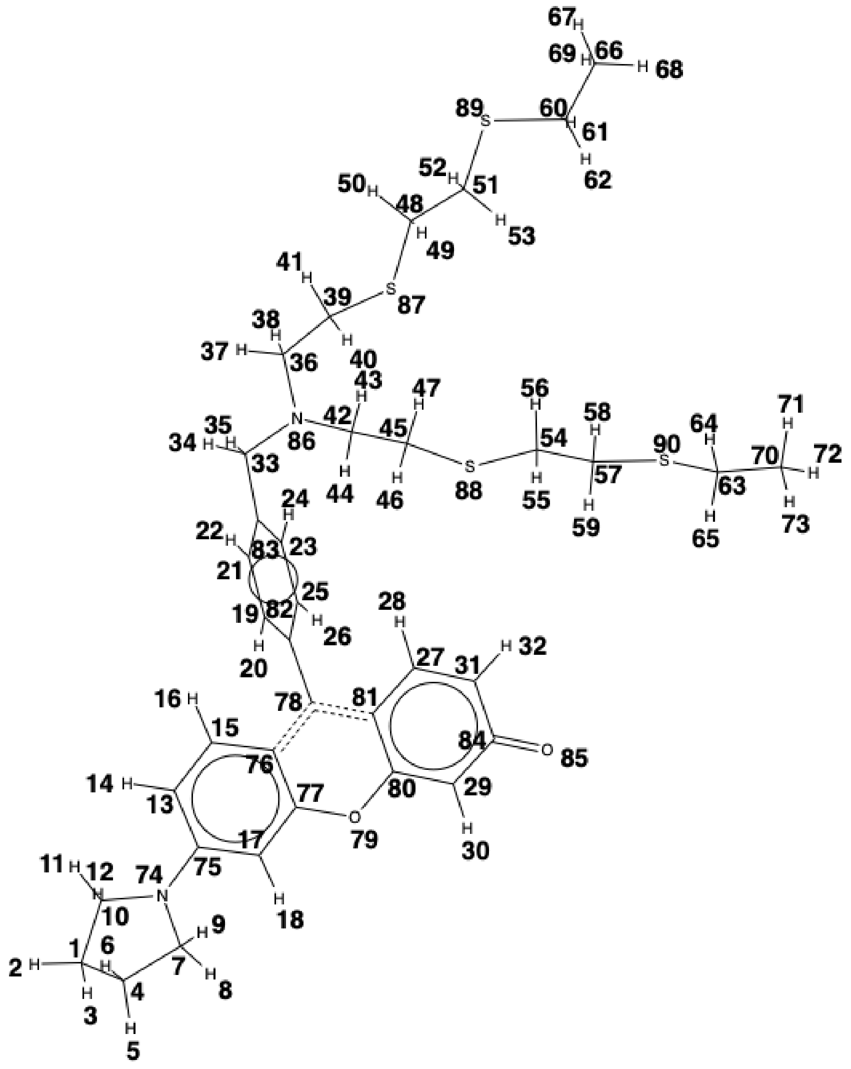

Figure S1: Molecule **1** atoms numbering.

Table S1: Maximum difference (MAX) between the force field optimized structural parameters and the four different exchange and correlation functionals ones, mean unsigned error (MUE) and root-mean-square deviation (RMSD) are shown at the bottom of the table. All distances in Å.

	FF	B3LYP	Δr	PBE0	Δr	BLYP	Δr	PBE	Δr
r(C1-H3)	1.099	1.098	0.001	1.099	0.001	1.099	0.001	1.098	0.002
r(C1-H2)	1.099	1.097	0.001	1.097	0.001	1.098	0.001	1.098	0.001
r(C1-C4)	1.541	1.547	0.007	1.545	0.005	1.543	0.003	1.546	0.005
r(C1-C10)	1.540	1.542	0.002	1.542	0.002	1.543	0.002	1.541	0.001
r(C4-H5)	1.099	1.097	0.002	1.097	0.002	1.099	0.000	1.099	0.000
r(C4-H6)	1.098	1.098	0.001	1.096	0.002	1.097	0.000	1.097	0.001
r(C4-C7)	1.532	1.541	0.009	1.539	0.008	1.538	0.006	1.541	0.009
r(C7-N74)	1.477	1.473	0.004	1.469	0.009	1.467	0.010	1.468	0.009
r(C7-H8)	1.097	1.099	0.003	1.098	0.001	1.097	0.001	1.099	0.002
r(C7-H9)	1.098	1.100	0.001	1.098	0.000	1.097	0.001	1.097	0.002
r(C10-H12)	1.097	1.098	0.001	1.098	0.001	1.097	0.001	1.098	0.000
r(C10-H11)	1.098	1.097	0.001	1.098	0.000	1.097	0.001	1.097	0.002
r(C10-N74)	1.467	1.461	0.006	1.459	0.007	1.460	0.006	1.460	0.007
r(C13-H14)	1.085	1.084	0.001	1.085	0.000	1.086	0.001	1.084	0.001
r(C13-C15)	1.409	1.403	0.006	1.405	0.004	1.406	0.003	1.401	0.008
r(C13-C75)	1.416	1.409	0.007	1.407	0.009	1.406	0.010	1.405	0.011
r(C15-C76)	1.401	1.405	0.004	1.406	0.004	1.402	0.000	1.405	0.003
r(C15-H16)	1.089	1.090	0.001	1.090	0.001	1.091	0.002	1.089	0.000
r(C17-H18)	1.083	1.083	0.000	1.083	0.000	1.083	0.000	1.082	0.000
r(C17-C75)	1.395	1.399	0.004	1.398	0.003	1.398	0.003	1.399	0.004
r(C17-C77)	1.392	1.393	0.001	1.393	0.001	1.392	0.000	1.396	0.004
r(C19-H20)	1.086	1.087	0.000	1.087	0.001	1.085	0.001	1.087	0.001

r(C19-C82)	1.412	1.412	0.000	1.413	0.001	1.413	0.001	1.414	0.002
r(C19-C21)	1.401	1.405	0.004	1.404	0.004	1.405	0.004	1.403	0.002
r(C21-H22)	1.085	1.086	0.001	1.086	0.001	1.086	0.001	1.087	0.002
r(C21-C83)	1.406	1.407	0.001	1.407	0.001	1.410	0.004	1.406	0.000
r(C23-C25)	1.401	1.401	0.001	1.407	0.005	1.403	0.002	1.406	0.005
r(C23-C83)	1.404	1.405	0.001	1.408	0.003	1.406	0.002	1.406	0.002
r(C23-H24)	1.087	1.086	0.001	1.086	0.001	1.086	0.001	1.087	0.000
r(C25-C82)	1.411	1.413	0.002	1.414	0.004	1.413	0.002	1.414	0.003
r(C25-H26)	1.087	1.087	0.001	1.087	0.000	1.088	0.000	1.086	0.002
r(C27-H28)	1.091	1.092	0.001	1.090	0.001	1.089	0.002	1.091	0.000
r(C27-C31)	1.405	1.403	0.002	1.405	0.000	1.404	0.002	1.404	0.001
r(C27-C81)	1.418	1.412	0.006	1.408	0.010	1.411	0.007	1.408	0.010
r(C29-H30)	1.082	1.082	0.000	1.083	0.000	1.083	0.001	1.084	0.001
r(C29-C80)	1.381	1.387	0.007	1.387	0.006	1.389	0.008	1.389	0.009
r(C29-C84)	1.390	1.393	0.003	1.393	0.003	1.393	0.003	1.395	0.005
r(C31-C84)	1.407	1.404	0.003	1.401	0.006	1.401	0.006	1.402	0.005
r(C31-H32)	1.086	1.082	0.004	1.083	0.003	1.087	0.000	1.085	0.002
r(C33-H34)	1.099	1.098	0.001	1.098	0.001	1.097	0.002	1.098	0.001
r(C33-H35)	1.099	1.097	0.002	1.098	0.001	1.098	0.001	1.099	0.000
r(C33-C83)	1.515	1.523	0.007	1.523	0.008	1.530	0.015	1.526	0.010
r(C33-N86)	1.486	1.482	0.004	1.487	0.001	1.480	0.006	1.487	0.001
r(C36-N86)	1.470	1.485	0.014	1.489	0.019	1.486	0.016	1.491	0.020
r(C36-H37)	1.096	1.098	0.002	1.098	0.002	1.098	0.002	1.097	0.001
r(C36-H38)	1.096	1.097	0.001	1.098	0.003	1.098	0.002	1.098	0.002
r(C36-C39)	1.547	1.561	0.014	1.546	0.001	1.564	0.017	1.549	0.002
r(C39-H41)	1.097	1.098	0.001	1.097	0.000	1.097	0.001	1.097	0.000

r(C39-S87)	1.864	1.858	0.006	1.840	0.024	1.855	0.009	1.845	0.019
r(C39-H40)	1.098	1.095	0.003	1.097	0.001	1.097	0.001	1.097	0.002
r(C42-N86)	1.489	1.502	0.014	1.508	0.019	1.497	0.008	1.488	0.001
r(C42-H43)	1.100	1.099	0.001	1.095	0.006	1.099	0.002	1.090	0.010
r(C42-H44)	1.097	1.097	0.000	1.097	0.000	1.096	0.001	1.098	0.001
r(C42-C45)	1.542	1.560	0.018	1.571	0.029	1.557	0.015	1.548	0.006
r(C45-S88)	1.855	1.861	0.006	1.872	0.017	1.857	0.002	1.840	0.015
r(C45-H46)	1.096	1.097	0.002	1.094	0.002	1.098	0.002	1.093	0.002
r(C45-H47)	1.095	1.095	0.000	1.096	0.001	1.095	0.000	1.098	0.003
r(C48-S87)	1.853	1.860	0.006	1.834	0.020	1.860	0.006	1.840	0.013
r(C48-H49)	1.098	1.098	0.000	1.097	0.001	1.098	0.000	1.098	0.000
r(C48-H50)	1.097	1.098	0.001	1.097	0.000	1.097	0.000	1.097	0.000
r(C48-C51)	1.545	1.545	0.001	1.531	0.015	1.547	0.002	1.535	0.011
r(C51-H52)	1.099	1.096	0.003	1.098	0.001	1.098	0.001	1.097	0.001
r(C51-S89)	1.858	1.863	0.006	1.834	0.023	1.858	0.000	1.844	0.013
r(C51-H53)	1.098	1.097	0.002	1.098	0.000	1.097	0.001	1.097	0.002
r(C54-S88)	1.851	1.869	0.018	1.875	0.024	1.866	0.015	1.850	0.000
r(C54-H56)	1.097	1.098	0.000	1.098	0.000	1.097	0.000	1.097	0.000
r(C54-C57)	1.542	1.558	0.016	1.567	0.025	1.552	0.010	1.535	0.007
r(C54-H55)	1.098	1.096	0.002	1.097	0.001	1.097	0.000	1.097	0.001
r(C57-H58)	1.097	1.097	0.000	1.099	0.002	1.098	0.000	1.098	0.001
r(C57-H59)	1.098	1.097	0.000	1.098	0.000	1.098	0.000	1.098	0.000
r(C57-S90)	1.842	1.874	0.032	1.869	0.027	1.866	0.024	1.843	0.001
r(C60-S89)	1.848	1.849	0.001	1.836	0.013	1.851	0.003	1.838	0.010
r(C60-H61)	1.098	1.098	0.000	1.098	0.000	1.098	0.000	1.098	0.000
r(C60-H62)	1.097	1.098	0.001	1.098	0.001	1.098	0.002	1.097	0.000

r(C60-C66)	1.549	1.544	0.006	1.541	0.009	1.539	0.010	1.540	0.009
r(C63-S90)	1.841	1.844	0.004	1.846	0.005	1.842	0.002	1.840	0.001
r(C63-H64)	1.098	1.097	0.001	1.097	0.001	1.098	0.001	1.098	0.001
r(C63-H65)	1.099	1.097	0.001	1.098	0.001	1.098	0.000	1.097	0.001
r(C63-C70)	1.543	1.543	0.000	1.544	0.000	1.536	0.007	1.542	0.001
r(C66-H69)	1.100	1.098	0.002	1.098	0.001	1.098	0.002	1.098	0.001
r(C66-H67)	1.098	1.097	0.000	1.097	0.001	1.097	0.000	1.098	0.000
r(C66-H68)	1.098	1.099	0.001	1.097	0.001	1.097	0.001	1.098	0.000
r(C70-H71)	1.097	1.098	0.001	1.098	0.001	1.098	0.001	1.097	0.000
r(C70-H72)	1.097	1.098	0.001	1.098	0.001	1.096	0.000	1.096	0.000
r(C70-H73)	1.097	1.098	0.002	1.098	0.002	1.097	0.000	1.098	0.001
r(N74-C75)	1.403	1.395	0.008	1.396	0.008	1.394	0.009	1.397	0.007
r(C74-C75)	1.401	1.403	0.002	1.405	0.004	1.406	0.005	1.404	0.003
r(C76-C78)	1.423	1.414	0.009	1.417	0.006	1.415	0.008	1.418	0.005
r(C77-O79)	1.356	1.363	0.007	1.359	0.003	1.362	0.006	1.359	0.003
r(C78-C82)	1.486	1.492	0.006	1.497	0.012	1.497	0.011	1.498	0.012
r(C78-C81)	1.415	1.415	0.000	1.417	0.002	1.415	0.000	1.419	0.003
r(O79-C80)	1.364	1.361	0.003	1.359	0.005	1.362	0.002	1.361	0.002
r(C80-C81)	1.405	1.404	0.001	1.406	0.001	1.404	0.002	1.410	0.005
r(C84-O85)	1.231	1.237	0.006	1.235	0.005	1.235	0.005	1.233	0.002
MAX		0.032		0.029		0.024		0.020	
MUE		0.004		0.005		0.004		0.004	
RMSD		0.006		0.009		0.006		0.006	

Table S2: Maximum difference (MAX) between the force field optimized structural parameters and the four different exchange and correlation functionals ones, mean unsigned error (MUE) and root-mean-square deviation (RMSD) are shown at the bottom of the table. All angles in degrees.

	FF	B3LYP	$\Delta\theta$	PBE0	$\Delta\theta$	BLYP	$\Delta\theta$	PBE	$\Delta\theta$
θ(H2-C1-H3)	107.81	108.52	0.71	108.46	0.65	108.77	0.96	108.61	0.80
θ(H3-C1-C4)	110.18	110.03	0.15	109.93	0.25	109.83	0.35	109.86	0.32
θ(H3-C1-C10)	109.97	108.99	0.98	109.33	0.65	109.29	0.69	109.32	0.66
θ(H2-C1-C4)	111.33	112.44	1.11	112.35	1.02	111.98	0.65	112.28	0.95
θ(H2-C1-C10)	110.83	112.07	1.23	112.11	1.27	112.37	1.54	112.09	1.26
θ(C4-C1-C10)	106.73	104.71	2.02	104.61	2.12	104.52	2.21	104.61	2.12
θ(C1-C4-H5)	111.50	112.62	1.12	112.26	0.75	112.43	0.93	112.41	0.90
θ(C1-C4-H6)	110.28	110.06	0.22	109.74	0.54	109.36	0.92	109.67	0.61
θ(C1-C4-C7)	105.85	104.29	1.56	104.49	1.36	104.43	1.42	104.49	1.36
θ(H5-C4-H6)	107.79	108.37	0.58	108.42	0.63	108.89	1.10	108.51	0.71
θ(C7-C4-H5)	111.54	112.47	0.93	112.49	0.96	112.79	1.25	112.46	0.92
θ(C7-C4-H6)	109.89	108.94	0.95	109.37	0.52	108.80	1.09	109.22	0.67
θ(C4-C7-N74)	106.77	105.51	1.27	105.79	0.99	105.79	0.98	105.80	0.97
θ(C4-C7-H8)	109.69	110.02	0.32	109.86	0.16	110.00	0.30	109.94	0.25
θ(C4-C7-H9)	109.98	111.74	1.76	111.52	1.54	111.64	1.66	111.38	1.39
θ(N74-C7-H8)	109.86	109.17	0.69	109.14	0.71	109.04	0.82	108.95	0.90
θ(N74-C7-H9)	111.07	111.11	0.04	110.77	0.30	110.80	0.27	110.92	0.15
θ(H8-C7-H9)	109.43	109.23	0.20	109.69	0.26	109.49	0.06	109.75	0.33
θ(C1-C10-H12)	109.63	112.13	2.50	111.81	2.18	111.78	2.15	111.52	1.89
θ(C1-C10-H11)	109.97	109.91	0.06	109.82	0.16	109.68	0.30	109.72	0.26
θ(C1-C10-N74)	106.98	105.68	1.30	105.86	1.12	105.94	1.04	106.13	0.85
θ(H11-C10-H12)	109.50	109.70	0.20	109.96	0.46	109.78	0.28	109.93	0.44

θ(N74-C10-H12)	110.43	110.63	0.19	110.51	0.08	110.65	0.22	110.50	0.07
θ(N74-C10-H11)	110.29	108.67	1.62	108.77	1.52	108.92	1.37	108.94	1.35
θ(C15-C13-H14)	118.79	119.10	0.31	119.23	0.45	119.24	0.45	119.01	0.22
θ(C75-C13-H14)	120.77	121.22	0.44	121.18	0.41	121.15	0.38	121.37	0.59
θ(C75-C13-C15)	120.44	119.68	0.76	119.59	0.86	119.60	0.84	119.62	0.82
θ(C13-C15-C76)	121.17	120.98	0.19	121.31	0.15	121.07	0.09	121.57	0.40
θ(C13-C15-H16)	119.60	119.48	0.11	119.00	0.60	119.19	0.41	118.37	1.23
θ(C76-C15-H16)	119.24	119.54	0.30	119.69	0.45	119.73	0.49	120.05	0.81
θ(C75-C17-H18)	121.43	121.49	0.06	121.75	0.31	121.74	0.31	121.63	0.20
θ(C77-C17-H18)	117.73	118.53	0.80	118.25	0.52	118.15	0.42	118.22	0.49
θ(C77-C17-C75)	120.84	119.98	0.86	120.01	0.83	120.11	0.73	120.15	0.69
θ(C82-C19-H20)	119.31	119.21	0.10	119.27	0.04	119.42	0.11	119.39	0.08
θ(C21-C19-H20)	119.78	120.05	0.27	119.69	0.10	119.65	0.13	119.52	0.26
θ(C82-C19-C21)	120.87	120.70	0.17	121.04	0.17	120.91	0.04	121.08	0.21
θ(C19-C21-H22)	119.88	119.52	0.36	119.54	0.34	119.73	0.15	119.71	0.17
θ(C19-C21-C83)	119.73	120.05	0.32	120.27	0.54	120.51	0.78	120.25	0.52
θ(C83-C21-H22)	120.33	120.39	0.06	120.19	0.14	119.75	0.58	120.04	0.30
θ(C83-C23-C25)	119.97	120.21	0.24	120.25	0.28	120.44	0.47	120.26	0.29
θ(C25-C23-H24)	120.08	119.86	0.22	119.93	0.16	119.65	0.43	119.91	0.17
θ(C83-C23-H24)	119.88	119.90	0.01	119.82	0.06	119.90	0.02	119.82	0.06
θ(C23-C25-C82)	120.67	120.80	0.13	120.90	0.23	121.14	0.47	120.93	0.25
θ(C23-C25-H26)	119.68	119.77	0.08	119.75	0.07	119.81	0.13	119.73	0.05
θ(C82-C25-H26)	119.59	119.40	0.20	119.35	0.25	119.04	0.56	119.34	0.25
θ(C31-C27-H28)	119.23	119.08	0.15	119.67	0.44	119.28	0.04	119.39	0.16
θ(C81-C27-H28)	121.29	121.51	0.21	120.98	0.32	121.06	0.23	120.98	0.31
θ(C81-C27-C31)	119.48	119.41	0.07	119.35	0.12	119.67	0.19	119.63	0.15

θ(C80-C29-H30)	118.87	119.51	0.65	119.52	0.65	119.49	0.62	119.79	0.93
θ(C84-C29-H30)	120.13	119.79	0.34	120.07	0.06	119.61	0.52	120.03	0.10
θ(C84-C29-C80)	121.00	120.68	0.32	120.41	0.59	120.90	0.10	120.18	0.82
θ(C27-C31-C84)	121.17	121.03	0.15	121.55	0.38	121.34	0.17	121.29	0.12
θ(C27-C31-H32)	120.05	119.52	0.53	119.98	0.07	119.62	0.42	119.91	0.14
θ(C84-C31-H32)	118.78	119.46	0.68	118.47	0.31	119.04	0.26	118.80	0.02
θ(H34-C33-H35)	107.43	107.67	0.24	106.68	0.75	107.47	0.04	106.95	0.49
θ(C83-C33-H34)	107.70	107.54	0.16	107.31	0.39	107.77	0.06	106.27	1.43
θ(N86-C33-H34)	109.26	109.37	0.11	109.23	0.03	109.65	0.39	109.51	0.25
θ(C83-C33-H35)	109.85	110.42	0.57	108.91	0.94	109.75	0.10	109.59	0.27
θ(N86-C33-H35)	110.02	109.93	0.09	109.05	0.97	109.54	0.48	109.21	0.81
θ(N86-C33-C83)	112.42	111.79	0.63	115.32	2.90	112.52	0.10	114.99	2.58
θ(N86-C36-H37)	108.78	107.82	0.96	109.20	0.42	108.27	0.51	109.26	0.48
θ(N86-C36-H38)	110.46	110.00	0.46	110.44	0.02	110.29	0.18	110.63	0.17
θ(N86-C36-C39)	114.27	115.19	0.92	113.50	0.77	114.57	0.30	113.19	1.09
θ(H37-C36-H38)	106.33	107.16	0.83	107.27	0.94	107.05	0.72	107.13	0.80
θ(C39-C36-H37)	106.45	106.76	0.31	106.39	0.05	106.76	0.32	106.28	0.17
θ(C39-C36-H38)	110.16	109.56	0.60	109.77	0.39	109.57	0.59	110.08	0.08
θ(C36-C39-H41)	105.97	107.46	1.49	109.78	3.82	107.78	1.82	109.32	3.35
θ(C36-C39-S87)	114.72	115.27	0.55	112.30	2.42	114.36	0.36	112.92	1.80
θ(C36-C39-H40)	111.70	110.98	0.72	110.06	1.65	110.77	0.93	109.71	1.99
θ(S87-C39-H41)	106.70	106.62	0.08	107.34	0.64	107.08	0.38	107.75	1.05
θ(H40-C39-H41)	106.37	106.71	0.34	107.28	0.91	106.95	0.57	107.21	0.83
θ(S87-C39-H40)	110.78	109.36	1.43	109.91	0.87	109.55	1.23	109.75	1.03
θ(N86-C42-H43)	110.61	110.03	0.59	110.50	0.12	110.42	0.20	109.12	1.49
θ(N86-C42-H44)	110.63	110.84	0.21	109.14	1.48	110.57	0.06	110.44	0.18

θ(N86-C42-C45)	111.12	113.11	1.98	115.32	4.19	112.85	1.73	113.71	2.59
θ(H43-C42-H44)	106.21	105.83	0.38	105.81	0.40	105.64	0.57	106.37	0.16
θ(C45-C42-H43)	108.43	108.74	0.30	106.56	1.87	108.67	0.24	107.49	0.94
θ(C45-C42-H44)	109.69	108.02	1.68	109.07	0.62	108.40	1.29	109.40	0.29
θ(C42-C45-S88)	110.49	110.30	0.19	111.42	0.93	110.41	0.08	110.43	0.06
θ(C42-C45-H46)	109.51	110.31	0.81	111.84	2.34	110.55	1.04	111.09	1.59
θ(C42-C45-H47)	109.72	110.48	0.76	109.23	0.49	109.82	0.10	110.82	1.10
θ(S88-C45-H46)	108.61	109.21	0.59	107.89	0.72	109.09	0.48	106.69	1.92
θ(S88-C45-H47)	111.00	109.11	1.89	109.27	1.72	109.09	1.91	109.41	1.59
θ(H46-C45-H47)	107.44	107.37	0.07	107.06	0.38	107.82	0.38	108.28	0.84
θ(S87-C48-H49)	109.38	109.18	0.21	109.12	0.26	109.74	0.36	109.64	0.26
θ(S87-C48-H50)	107.92	109.60	1.68	109.91	1.98	109.80	1.88	109.32	1.40
θ(S87-C48-C51)	114.53	110.76	3.77	109.65	4.88	110.01	4.52	109.91	4.62
θ(H49-C48-H50)	107.36	107.87	0.51	108.05	0.69	107.68	0.32	108.08	0.72
θ(C51-C48-H49)	109.80	109.69	0.10	109.32	0.48	109.82	0.02	109.40	0.40
θ(C51-C48-H50)	107.58	109.68	2.10	110.76	3.18	109.75	2.17	110.46	2.88
θ(C48-C51-H52)	110.61	110.36	0.25	110.64	0.03	110.09	0.52	110.38	0.23
θ(C48-C51-S89)	107.81	112.92	5.12	109.34	1.53	112.70	4.89	110.25	2.45
θ(C48-C51-H53)	110.01	108.44	1.57	109.95	0.06	108.48	1.53	109.36	0.65
θ(C48-C51-H52)	109.85	108.77	1.08	110.20	0.35	108.81	1.04	109.63	0.22
θ(H52-C51-H53)	108.21	106.99	1.22	107.89	0.32	107.26	0.95	107.63	0.58
θ(S89-C51-H53)	110.36	109.18	1.18	108.79	1.57	109.35	1.00	109.55	0.81
θ(S88-C54-H56)	109.19	109.67	0.48	108.86	0.33	109.82	0.63	110.68	1.49
θ(S88-C54-C57)	111.28	109.16	2.12	112.20	0.92	109.26	2.02	109.79	1.49
θ(S88-C54-H55)	109.21	109.37	0.16	109.11	0.10	109.96	0.75	109.53	0.32
θ(C57-C54-H56)	109.77	109.93	0.16	109.75	0.02	109.87	0.09	108.81	0.96

θ(H55-C54-H56)	107.81	107.98	0.18	107.35	0.45	107.81	0.00	107.60	0.20
θ(C57-C54-H55)	109.51	110.71	1.19	109.45	0.07	110.11	0.60	110.40	0.89
θ(C54-C57-H58)	110.08	108.77	1.31	109.71	0.37	109.10	0.98	109.69	0.39
θ(C54-C57-H59)	110.28	108.64	1.64	109.52	0.77	109.12	1.16	110.09	0.19
θ(C54-C57-S90)	109.47	115.38	5.91	112.87	3.40	113.88	4.41	110.33	0.86
θ(H58-C57-H59)	107.87	106.87	1.00	107.20	0.67	107.13	0.74	107.85	0.03
θ(S90-C57-H58)	109.72	108.63	1.09	108.70	1.02	109.09	0.63	109.05	0.67
θ(S90-C57-H57)	109.39	108.23	1.16	108.68	0.71	108.31	1.08	109.79	0.40
θ(S89-C60-H61)	109.04	109.25	0.21	109.02	0.02	109.61	0.57	109.20	0.16
θ(S89-C60-H62)	109.46	108.55	0.91	108.89	0.57	109.19	0.27	108.41	1.05
θ(S89-C60-C66)	110.39	111.10	0.71	110.15	0.24	110.61	0.22	110.78	0.39
θ(H61-C60-H62)	107.86	107.61	0.25	108.13	0.27	107.80	0.06	108.09	0.23
θ(C66-C60-H61)	110.15	110.30	0.15	110.18	0.03	109.86	0.29	110.18	0.03
θ(C66-C60-H62)	109.89	109.95	0.06	110.42	0.53	109.72	0.18	110.12	0.23
θ(S90-C63-H64)	108.78	108.64	0.13	108.95	0.17	109.21	0.44	109.10	0.33
θ(S90-C63-H65)	108.80	109.74	0.94	108.92	0.12	109.45	0.65	109.05	0.25
θ(S90-C63-C70)	110.86	109.89	0.97	110.70	0.16	109.76	1.10	110.87	0.01
θ(H64-C63-H65)	108.29	107.90	0.39	108.09	0.20	108.10	0.19	107.85	0.44
θ(C70-C63-H64)	109.75	110.12	0.37	110.35	0.61	110.12	0.38	109.82	0.07
θ(C70-C63-H65)	110.31	110.51	0.20	109.78	0.53	110.16	0.15	110.09	0.22
θ(C60-C66-H69)	110.69	110.93	0.24	110.65	0.04	110.57	0.12	110.59	0.09
θ(C60-C66-H67)	110.55	110.55	0.00	110.49	0.06	110.55	0.01	110.40	0.15
θ(C60-C66-H68)	111.21	110.56	0.65	110.57	0.64	110.71	0.50	110.62	0.59
θ(C60-C66-H67)	108.24	107.95	0.29	108.32	0.08	108.26	0.03	108.42	0.18
θ(H68-C66-H69)	108.00	108.26	0.25	108.32	0.31	108.22	0.22	108.28	0.28
θ(H67-C66-H68)	108.03	108.50	0.47	108.42	0.39	108.43	0.41	108.44	0.42

θ(C63-C70-H71)	110.23	110.02	0.21	110.79	0.56	110.33	0.10	110.50	0.27
θ(C63-C70-H72)	110.72	110.98	0.26	110.44	0.28	110.52	0.20	110.62	0.11
θ(C63-C70-H73)	111.01	110.86	0.15	110.52	0.49	110.64	0.37	110.89	0.12
θ(H71-C70-H72)	108.36	108.51	0.15	108.44	0.08	108.64	0.29	108.01	0.35
θ(H71-C70-H73)	108.00	108.11	0.11	108.08	0.08	108.15	0.15	108.45	0.45
θ(H72-C70-H73)	108.43	108.28	0.15	108.48	0.05	108.48	0.05	108.28	0.14
θ(C7-N74-C10)	110.89	111.91	1.02	111.82	0.92	111.63	0.74	111.69	0.80
θ(C7-N74-C75)	124.46	124.40	0.07	124.58	0.12	124.20	0.26	124.69	0.23
θ(C10-N74-C75)	123.83	123.65	0.17	123.60	0.23	124.16	0.33	123.61	0.22
θ(C13-C75-C17)	118.05	119.63	1.58	119.64	1.59	119.67	1.62	119.48	1.42
θ(C13-C75-N74)	121.89	121.05	0.85	120.84	1.06	120.94	0.96	120.41	1.48
θ(C17-C75-N74)	120.05	119.32	0.73	119.52	0.53	119.39	0.66	120.11	0.06
θ(C15-C76-C77)	117.38	118.28	0.90	117.77	0.39	118.22	0.84	117.72	0.34
θ(C15-C76-C78)	122.86	121.71	1.15	122.17	0.68	121.89	0.97	122.00	0.86
θ(C77-C76-C78)	119.76	120.00	0.24	120.06	0.30	119.87	0.11	120.27	0.51
θ(C17-C77-C76)	122.11	121.42	0.69	121.68	0.43	121.32	0.79	121.45	0.66
θ(C17-C77-O79)	117.13	117.95	0.82	117.53	0.40	117.88	0.75	117.89	0.75
θ(C76-C77-O79)	120.75	120.62	0.14	120.78	0.03	120.79	0.03	120.66	0.10
θ(C76-C78-C82)	122.15	121.83	0.33	121.30	0.85	121.69	0.47	120.13	2.02
θ(C76-C78-C81)	118.17	118.06	0.11	117.67	0.50	117.85	0.32	117.62	0.56
θ(C82-C78-C81)	119.66	120.01	0.35	121.02	1.36	120.32	0.66	122.24	2.58
θ(C77-O79-C80)	120.96	120.68	0.28	120.71	0.26	120.70	0.26	120.84	0.12
θ(O79-C80-C29)	117.42	117.92	0.51	117.32	0.09	118.02	0.60	117.52	0.10
θ(C81-C80-C29)	121.53	120.97	0.55	121.62	0.10	121.17	0.36	121.54	0.02
θ(O79-C80-C81)	121.05	121.08	0.03	121.05	0.00	120.80	0.25	120.94	0.12
θ(C78-C81-C27)	122.47	121.56	0.92	121.87	0.60	121.59	0.88	121.96	0.51

θ(C80-C81-C27)	118.25	118.91	0.66	118.42	0.17	118.43	0.19	118.39	0.14
θ(C78-C81-C80)	119.28	119.53	0.25	119.71	0.43	119.97	0.70	119.64	0.36
θ(C19-C82-C25)	118.68	118.58	0.10	118.19	0.49	118.04	0.64	118.09	0.59
θ(C19-C82-C78)	120.30	121.33	1.03	120.36	0.06	121.94	1.64	120.63	0.33
θ(C25-C82-C78)	120.70	119.95	0.75	121.44	0.74	119.95	0.75	121.18	0.48
θ(C21-C83-C23)	120.01	119.62	0.38	119.35	0.66	118.95	1.06	119.37	0.64
θ(C21-C83-C33)	120.56	121.19	0.63	120.04	0.52	120.45	0.11	120.03	0.54
θ(C23-C83-C33)	119.37	119.12	0.25	120.60	1.23	120.59	1.22	120.60	1.23
θ(C31-C84-C29)	118.56	118.92	0.36	118.63	0.06	118.46	0.11	118.95	0.38
θ(C29-C84-O85)	120.25	120.54	0.28	120.48	0.23	120.60	0.35	120.66	0.41
θ(C31-C84-O85)	121.18	120.54	0.64	120.89	0.29	120.94	0.24	120.39	0.79
θ(C33-N86-C36)	112.22	112.22	0.01	108.96	3.26	113.41	1.19	110.13	2.08
θ(C33-N86-C42)	116.76	114.04	2.72	113.29	3.47	113.27	3.50	113.36	3.40
θ(C36-N86-C42)	113.75	114.57	0.83	114.23	0.49	114.49	0.74	113.80	0.05
θ(C39-S87-C48)	98.53	100.23	1.71	98.84	0.31	102.01	3.48	99.78	1.26
θ(C45-S88-C54)	101.30	104.85	3.55	105.10	3.81	104.55	3.25	101.10	0.20
θ(C51-S89-C60)	102.75	99.80	2.95	98.96	3.78	99.89	2.86	99.59	3.16
θ(C57-S90-C63)	100.78	100.74	0.05	101.54	0.76	100.62	0.16	99.13	1.65
MAX		5.91		4.88		4.89		4.62	
MUE		0.72		0.74		0.73		0.72	
RMSD		1.11		1.16		1.10		1.05	

The bond lengths and angles calculated with the B3LYP, PBE0, BLYP and PBE functionals giving the maximum difference with respect the FF ones are, respectively, r(C57-S90), r(C42-C45), r(C57-S90), r(C36-N86) and θ(C54-C57-S90), θ(S87-C48-C51), θ(C48-C51-S89), θ(S87-C48-C51). Since none of these structural parameters are involved in the

fluorophore part of the molecule, we can assume that the chosen force field is reliable.

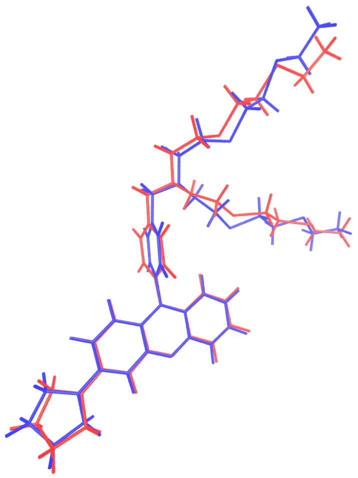

(a) *B3LYP.*

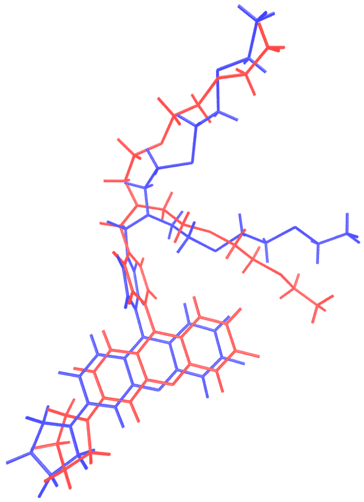

(b) *PBE0.*

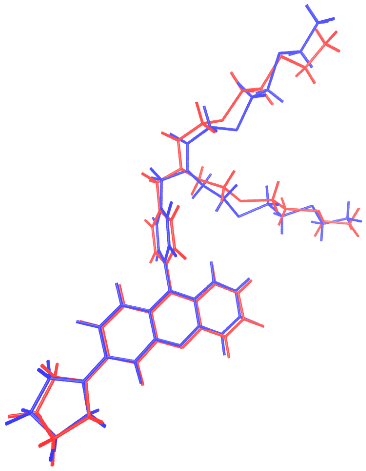

(c) *BLYP.*

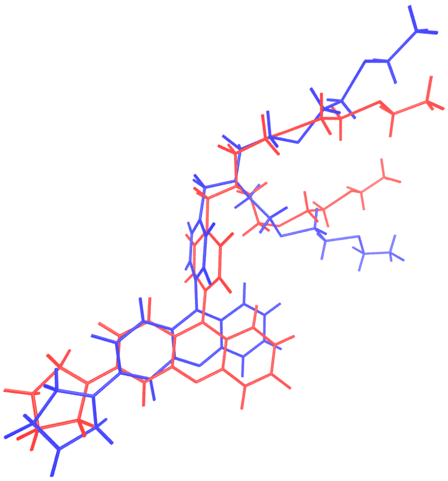

(d) *PBE.*

Figure S2: Comparison between the isolated molecule **1** FF-optimized (blue) and optimized with the four different exchange and correlation functionals (red), specified in every caption.

S2 Structural analysis derived from MD simulations of the molecules 1 and Meth-1 in the gas phase and in solution

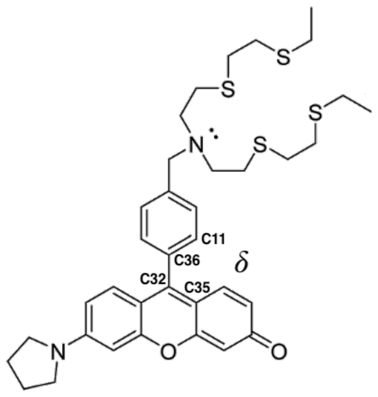

(a) *Analysis of the dihedral angle δ (C11-C36-C32-C35).*

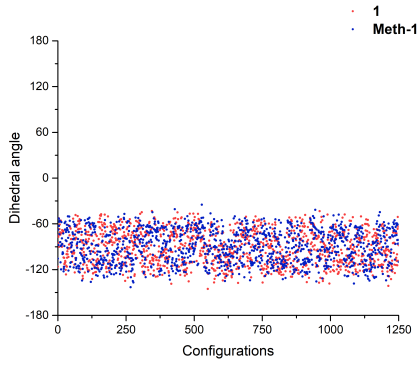

(b) *Comparison between the values of the dihedral angle δ for the molecules **1** and **Meth-1** in the gas phase.*

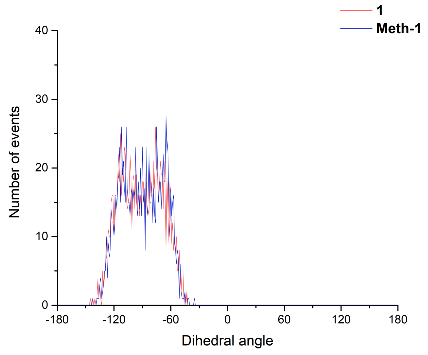

(c) *Comparison between the distribution of the dihedral angle δ for the molecules **1** and **Meth-1** in the gas phase with a step of 1 degree.*

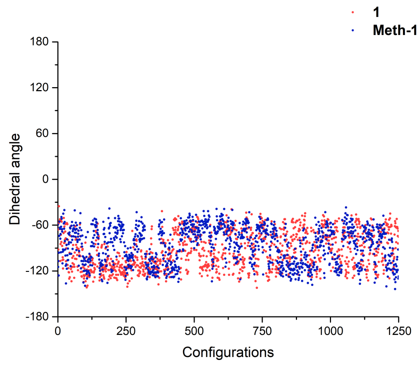

(d) *Comparison between the values of the dihedral angle δ for the molecules **1** and **Meth-1** in solution.*

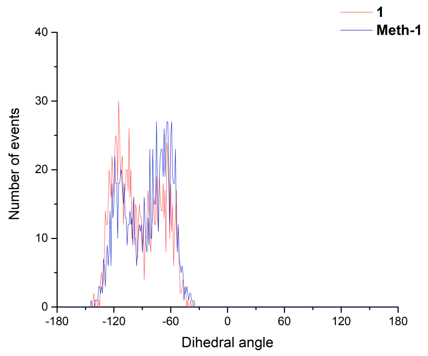

(e) *Comparison between the values distribution of the dihedral angle δ for the molecules **1** and **Meth-1** in solution with a step of 1 degree.*

Figure S3: Comparison between the values and the values distribution of the dihedral angle δ for the molecules **1** and **Meth-1** both in the gas phase and in solution.

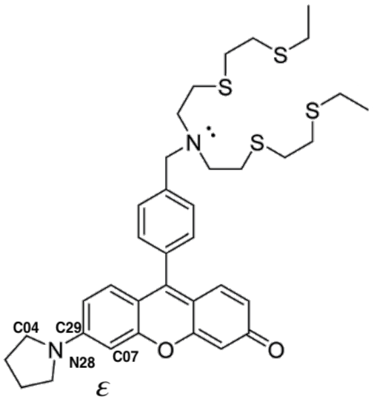

(a) *Analysis of the dihedral angle* ε *(C04-N28-C29-C07).*

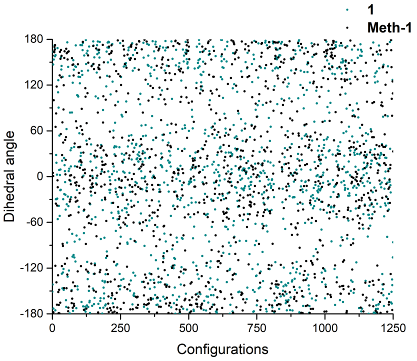

(b) *Comparison between the values of the dihedral angle* ε *for the molecules* ***1*** *and* ***Meth-1*** *in the gas phase.*

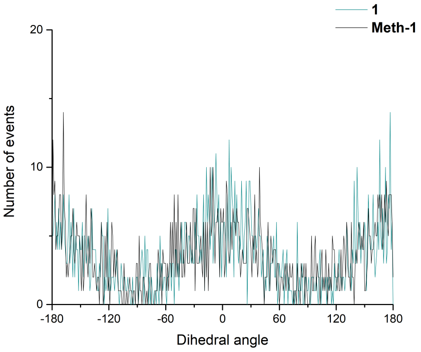

(c) *Comparison between the values distribution of the dihedral angle* ε *for the molecules* ***1*** *and* ***Meth-1*** *in the gas phase with a step of 1 degree.*

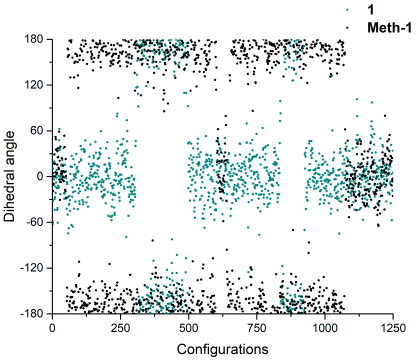

(d) *Comparison between the values of the dihedral angle* ε *for the molecules* ***1*** *and* ***Meth-1*** *in solution.*

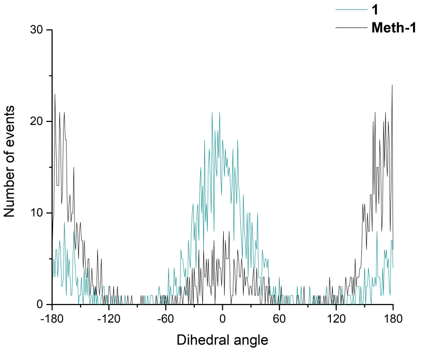

(e) *Comparison between the values distribution of the dihedral angle* ε *for the molecules****1*** *and* ***Meth-1*** *in solution with a step of 1 degree.*

Figure S4: Comparison between the values and the values distribution of the dihedral angle ε for the molecules **1** and **Meth-1** both in the gas phase and in solution.

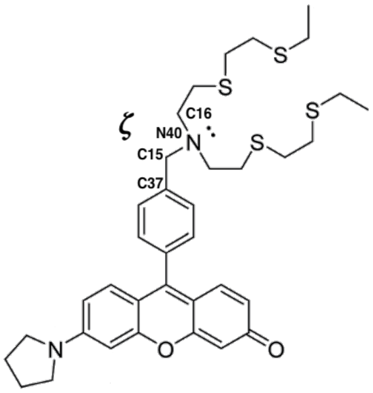

(a) *Analysis of the dihedral angle ζ (C16-N40-C15-C37).*

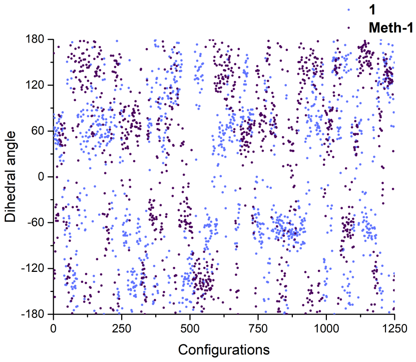

(b) *Comparison between the values of the dihedral angle ζ for the molecules **1** and **Meth-1** in the gas phase.*

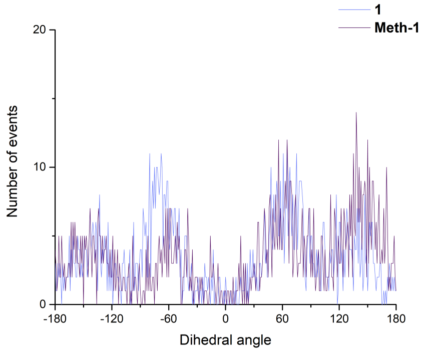

(c) *Comparison between the values distribution of the dihedral angle ζ for the molecules **1** and **Meth-1** in the gas phase with a step of 1 degree.*

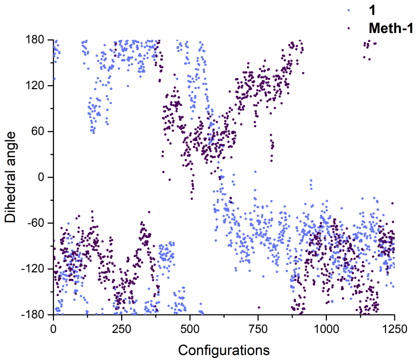

(d) *Comparison between the values of the dihedral angle ζ for the molecules **1** and **Meth-1** in solution.*

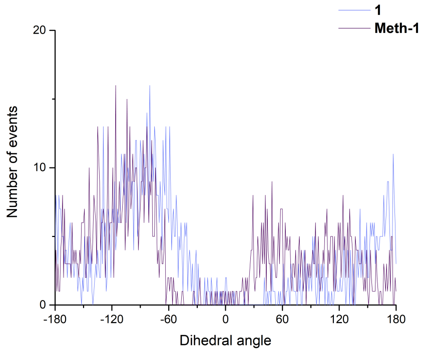

(e) *Comparison between the values distribution of the dihedral angle ζ for the molecules **1** and **Meth-1** in solution with a step of 1 degree.*

Figure S5: Comparison between the values and the values distribution of the dihedral angle ζ for the molecules **1** and **Meth-1** both in the gas phase and in solution.

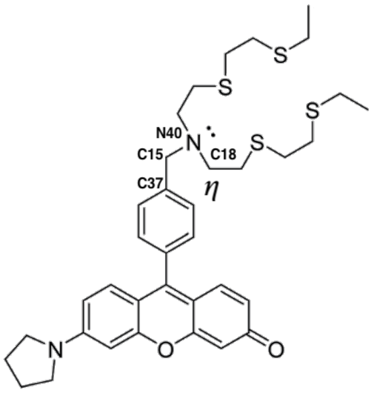

(a) *Analysis of the dihedral angle* η *(C18-N40-C15-C37).*

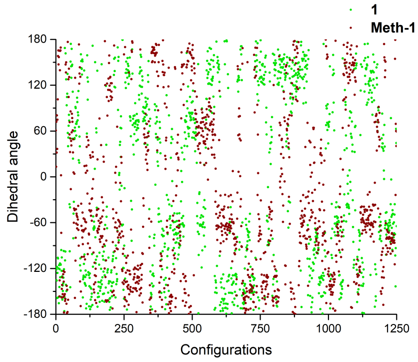

(b) *Comparison between the values of the dihedral angle* η *for the molecules* ***1*** *and* ***Meth-1*** *in the gas phase.*

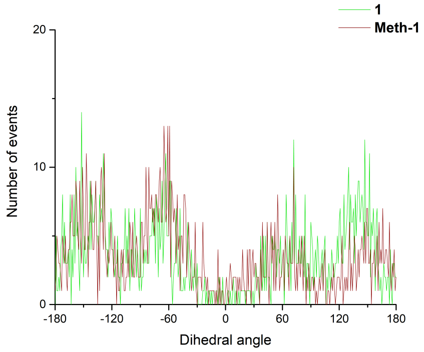

(c) *Comparison between the values distribution of the dihedral angle* η *for the molecules* ***1*** *and* ***Meth-1*** *in the gas phase with a step of 1 degree.*

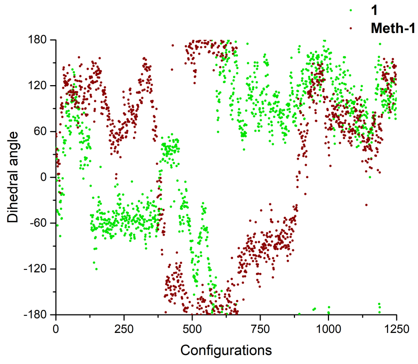

(d) *Comparison between the values of the dihedral angle* η *for the molecules* ***1*** *and* ***Meth-1*** *in solution.*

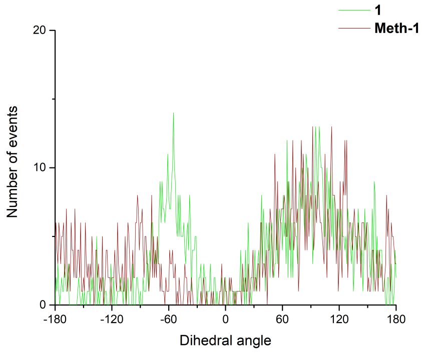

(e) *Comparison between the values distribution of the dihedral angle* η *for the molecules* ***1*** *and* ***Meth-1*** *in solution with a step of 1 degree.*

Figure S6: Comparison between the values and the values distribution of the dihedral angle η for the molecules **1** and **Meth-1** both in the gas phase and in solution.

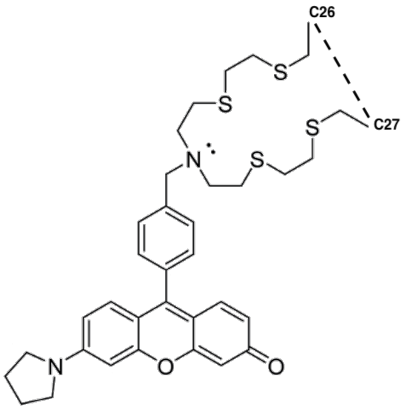

(a) *Analysis of the distance alkyl inter-chains distance (C27-C26).*

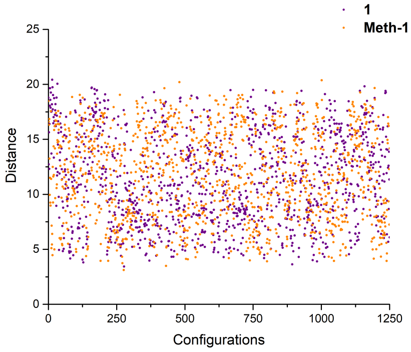

(b) *Comparison between the values of the distance C27-C26 for the molecules* ***1*** *and* ***Meth-1*** *in the gas phase.*

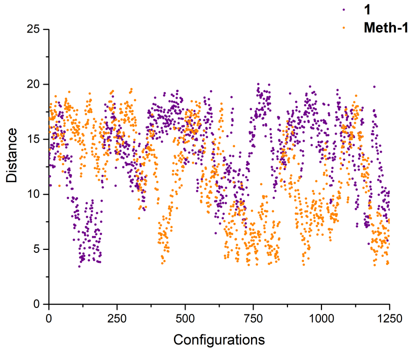

(c) *Comparison between the distribution of values of the distance C27-C26 for the molecules* ***1*** *and* ***Meth-1*** *in solution.*

Figure S7: Comparison between the values of the distance C27-C26 for the molecules **1** and **Meth-1** both in the gas phase and in solution.

From the analysis of the mobility of the two molecules it was observed that these are extremely similar, therefore comparable as regards the conformational variation during the Molecular Dynamics simulation, both in the gas phase and in solution.

S3 DFT and TD-DFT results: molecular orbitals analysis, detailed transition energies and optimized structures

The data obtained at DFT and TD-DFT level for **1** and **Meth-1** will be here reported for each of the four functional considered in the gas phase and in solution.

S3.1 PBE0 results in the gas phase

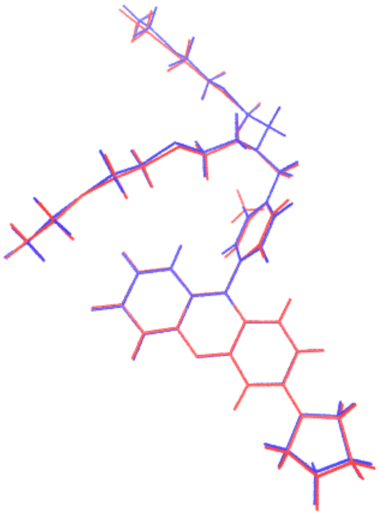

Figure S8: Comparison between the optimized structures of the molecules **1** (blue) and **Meth-1** (red) calculated using the PBE0 functional in the gas phase.

Table S3: Excitation energy (E, in eV, and λ in nm), oscillator strength (f) and molecular orbitals involved in each electronic transition computed at PBE0 level for the molecules 1 and Meth-1 in the gas phase.

		**1**	**Meth-1**
Excited state 1	E (eV)	2.8906	2.9057
	λ (nm)	428.93	426.70
	f	0.0060	0.0113
	MOs (c)	174 → 179 (0.59842)	178 → 183 (0.61168)
		176 → 179 (0.17745)	180 → 183 (0.15458)
		177 → 179 (-0.27194)	181 → 183 (-0.25564)
			182 → 183 (0.10307)
Excited state 2	E (eV)	2.9359	2.9488
	λ (nm)	422.30	420.45
	f	0.6277	0.6299
	MOs (c)	178 → 179 (0.68377)	178 → 183 (-0.10021)
			182 → 183 (0.68007)
Excited state 3	E (eV)	3.1192	3.1187
	λ (nm)	397.48	397.55
	f	0.0009	0.0025
	MOs (c)	174 → 179 (0.26739)	178 → 183 (0.25206)
		177 → 179 (0.64966)	181 → 183 (0.65658)
Excited state 4	E (eV)	3.4153	3.4068
	λ (nm)	363.02	363.94
	f	0.0021	0.0005
	MOs (c)	173 → 179 (0.10405)	178 → 183 (-0.16361)
		174 → 179 (-0.19292)	179 → 183 (-0.12720)
		175 → 179 (0.17465)	180 → 183 (0.66927)
		176 → 179 (0.64229)	
Excited state 5	E (eV)	3.4591	3.4752
	λ (nm)	358.43	356.77
	f	0.1007	0.1063
	MOs (c)	170 → 179 (0.13595)	174 → 183 (0.12838)
		172 → 179 (-0.34198)	177 → 183 (0.67239)
		173 → 179 (0.56996)	
		176 → 179 (-0.11601)	

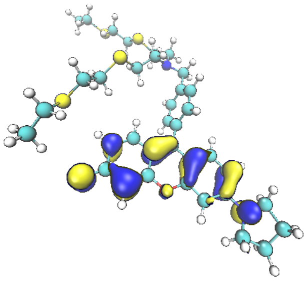

(a) ***1** molecular orbital 178 (HOMO).*

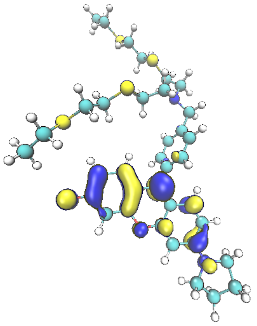

(b) ***1** molecular orbital 179 (LUMO).*

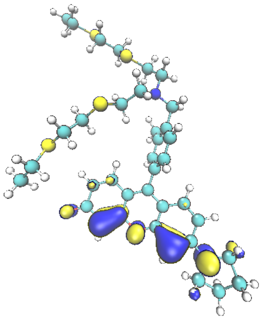

(c) ***Meth-1** molecular orbital 178.*

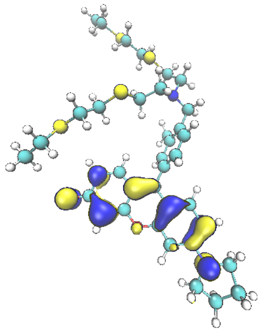

(d) ***Meth-1** molecular orbital 182 (HOMO).*

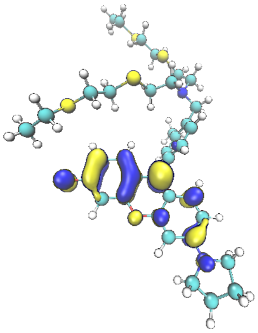

(e) ***Meth-1** molecular orbital 183 (LUMO).*

Figure S9: Most relevant orbitals of the molecules **1** and **Meth-1** computed using the PBE0 functional in the gas phase.

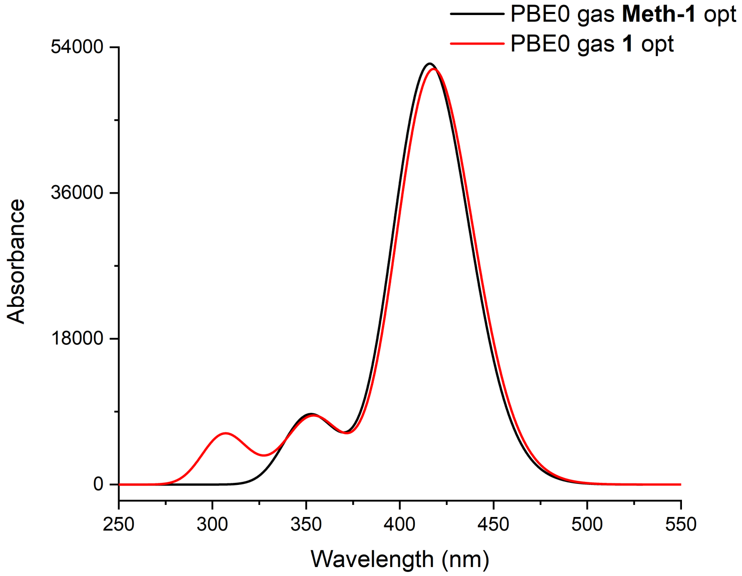

Figure S10: Comparison between the absorption spectra of the molecules **1** and **Meth-1** computed using the PBE0 functional in the gas phase.

S3.2 PBE0 results in solution

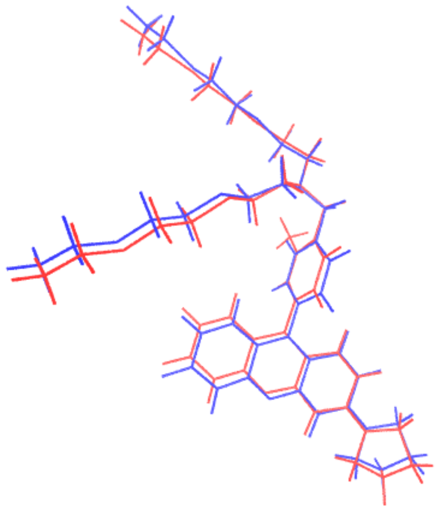

Figure S11: Comparison between the optimized structures of the molecules **1** (blue) and **Meth-1** (red) calculated using the PBE0 functional in solution.

Table S4: Excitation energy (E, in eV, and λ in nm), oscillator strength (f) and molecular orbitals involved in each electronic transition computed at PBE0 level for the molecules 1 and Meth-1 in solution.

		**1**	**Meth-1**
Excited state 1	E (eV)	2.5453	2.5467
	λ (nm)	487.10	486.85
	f	1.2446	1.2418
	MOs (c)	178 → 179 (0.70388)	182 → 183 (0.70387)
Excited state 2	E (eV)	3.3012	3.3049
	λ (nm)	375.58	375.15
	f	0.0483	0.0443
	MOs (c)	170 → 179 (-0.11773)	174 → 183 (0.11017)
		174 → 179 (0.63687)	177 → 183 (0.11890)
		175 → 179 (-0.17110)	178 → 183 (0.65882)
		177 → 179 (-0.17852)	181 → 183 (-0.13266)
Excited state 3	E (eV)	3.3346	3.3346
	λ (nm)	371.82	371.81
	f	0.0039	0.0028
	MOs (c)	171 → 179 (0.60879)	175 → 183 (0.53312)
		174 → 179 (-0.15479)	178 → 183 (-0.12966)
		175 → 179 (-0.12511)	179 → 183 (-0.10868)
		176 → 179 (0.10957)	180 → 183 (-0.11893)
		177 → 179 (-0.24073)	181 → 183 (-0.38955)
Excited state 4	E (eV)	3.3765	3.3678
	λ (nm)	367.20	368.15
	f	0.0095	0.0006
	MOs (c)	170 → 179 (-0.13663)	174 → 183 (0.16901)
		171 → 179 (0.28466)	175 → 183 (0.39885)
		174 → 179 (0.17167)	180 → 183 (0.17755)
		175 → 179 (0.29143)	181 → 183 (0.50696)
		177 → 179 (0.52171)	
Excited state 5	E (eV)	3.4044	3.4168
	λ (nm)	364.19	362.86
	f	0.0024	0.0002
	MOs (c)	175 → 179 (0.14430)	179 → 183 (-0.48677)
		176 → 179 (0.67302)	180 → 183 (0.50502)

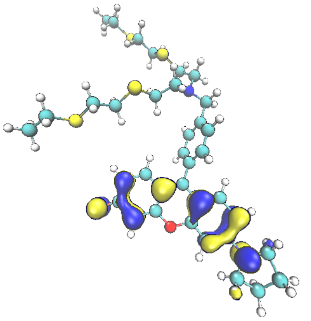

(a) ***1*** *molecular orbital 178 (HOMO).*

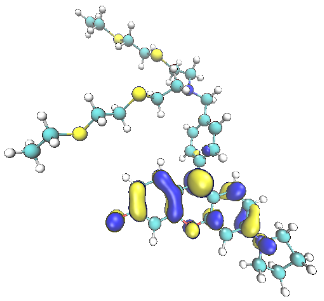

(b) ***1*** *molecular orbital 179 (LUMO).*

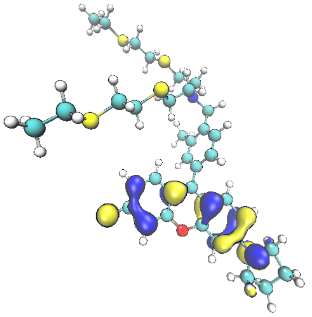

(c) ***Meth-1*** *molecular orbital 182 (HOMO).*

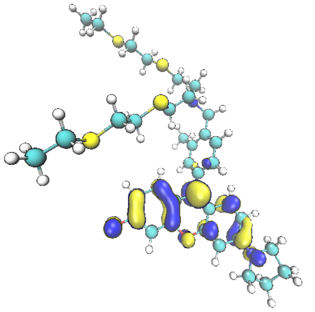

(d) ***Meth-1*** *molecular orbital 183 (LUMO).*

Figure S12: Most relevant orbitals of the molecules **1** and **Meth-1** computed using the PBE0 functional in solution.

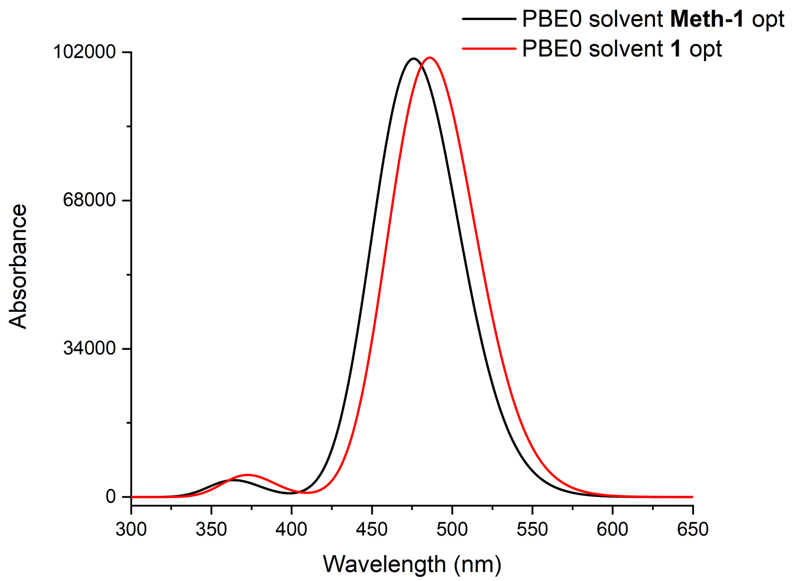

Figure S13: Comparison between the absorption spectra of the molecules **1** and **Meth-1** computed using the PBE0 functional in solution.

S3.3 PBE results in the gas phase

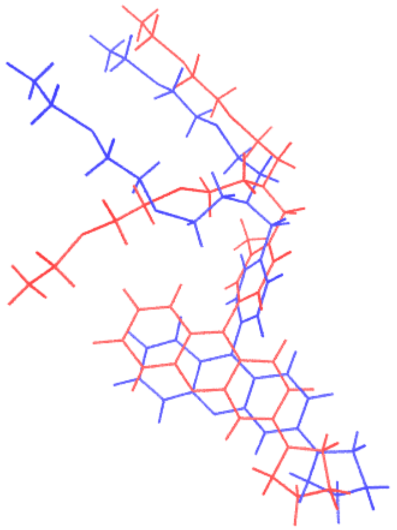

Figure S14: Comparison between the optimized structures of the molecules **1** (blue) and **Meth-1** (red) calculated using the PBE functional in the gas phase.

As it can be seen in Figure S14, the optimized structures of **1** (blue) and **Meth-1** (red) differ essentially for the orientation of the thioalkyl chains, that are actually corresponding to two different local minima. Re-optimization of **Meth-1** starting from the optimized conformation of **1** or re-optimization of **1** starting from the local minima obtained for **Meth-1** lead to two other minima separated by a less than 0.2 kcal/mol from those previously optimized with identical chain conformation, confirming the high mobility of these substituents. Nonetheless, the computed spectroscopic features are not affected by this parameter.

Table S5: Excitation energy (E, in eV, and λ in nm), oscillator strength (f) and molecular orbitals involved in each electronic transition computed at PBE level for the molecules 1 and Meth-1 in the gas phase.

		**1**	**Meth-1**
Excited state 1	E (eV)	1.8260	1.7678
	λ (nm)	679.00	701.35
	f	0.0001	0.0001
	MOs (c)	178 → 179 (0.70664)	180 → 183 (0.25528)
			182 → 183 (0.65927)
Excited state 2	E (eV)	2.0448	1.8952
	λ (nm)	606.34	654.20
	f	0.0000	0.0000
	MOs (c)	176 → 179 (0.70706)	180 → 183 (0.62917)
			181 → 183 (-0.20895)
			182 → 183 (-0.24094)
Excited state 3	E (eV)	2.1303	2.0545
	λ (nm)	582.00	603.49
	f	0.0001	0.0004
	MOs (c)	175 → 179 (0.70705)	179 → 183 (0.70541)
Excited state 4	E (eV)	2.2657	2.1655
	λ (nm)	547.21	572.53
	f	0.0002	0.0000
	MOs (c)	174 → 179 (0.70694)	178 → 183 (0.70706)
Excited state 5	E (eV)	2.4068	2.2607
	λ (nm)	515.13	548.43
	f	0.0005	0.0000
	MOs (c)	173 → 179 (0.70649)	177 → 183 (0.70691)
Excited state 6	E (eV)	2.5037	2.4552
	λ (nm)	495.21	504.99
	f	0.3413	0.0000

	MOs (c)	170 → 179 (0.13458)<br>172 → 179 (0.13236)<br>177 → 179 (0.65309)<br>177 → 180 (0.13466)<br>177 ← 179 (-0.10758)	176 → 183 (0.70704)
Excited state 7	E (eV)	2.5320	2.5375
	λ (nm)	489.68	488.60
	f	0.0159	0.4156
	MOs (c)	171 → 179 (0.68981)<br>172 → 179 (-0.11005)<br>177 → 179 (0.10309)	174 → 183 (0.12449)<br>175 → 183 (0.11861)<br>180 → 183 (0.18217)<br>181 → 183 (0.63741)<br>181 ← 183 (-0.10568)
Excited state 8	E (eV)	2.7400	2.7674
	λ (nm)	452.49	448.02
	f	0.0934	0.1092
	MOs (c)	171 → 179 (0.12573)<br>172 → 179 (0.66285)<br>177 → 179 (-0.10316)<br>177 → 180 (-0.11940)	175 → 183 (0.67648)<br>181 → 183 (-0.11065)
Excited state 9	E (eV)	2.9387	3.0097
	λ (nm)	421.90	411.94
	f	0.0009	0.0004
	MOs (c)	178 → 180 (0.70601)	182 → 184 (0.70592)
Excited state 10	E (eV)	3.0671	3.1034
	λ (nm)	404.24	399.51
	f	0.0913	0.0013
	MOs (c)	177 → 179 (-0.12548)<br>177 → 180 (0.66577)<br>177 → 182 (0.12748)	180 → 184 (0.66242)<br>181 → 184 (-0.24433)

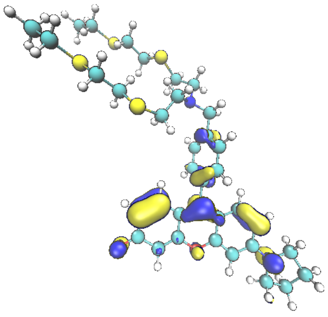

(a) ***1*** *molecular orbital 170.*

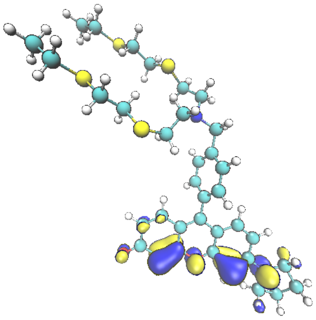

(b) ***1*** *molecular orbital 172.*

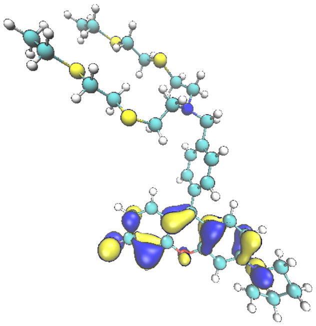

(c) ***1*** *molecular orbital 177.*

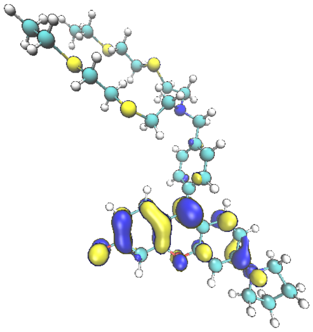

(d) ***1*** *molecular orbital 179 (LUMO).*

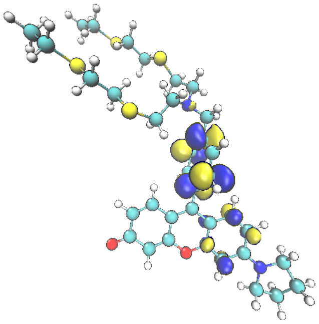

(e) ***1*** *molecular orbital 180.*

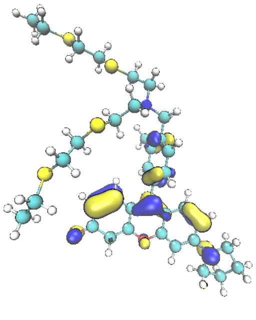

(f) ***Meth-1*** *molecular orbital 174.*

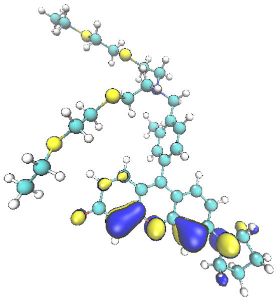

(g) ***Meth-1*** *molecular orbital 175.*

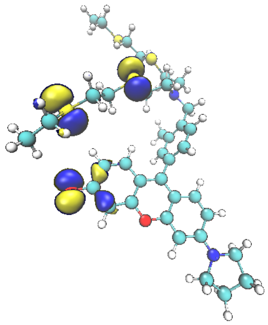

(h) ***Meth-1*** *molecular orbital 180.*

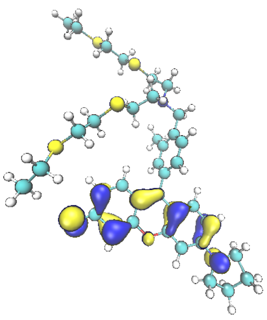

(i) ***Meth-1*** *molecular orbital 181.*

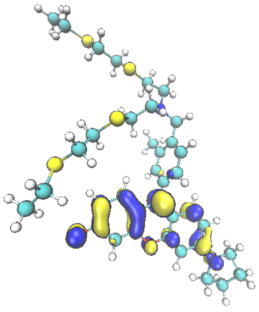

(j) ***Meth-1*** *molecular orbital 183 (LUMO).*

Figure S15: Most relevant orbitals of the molecules **1** and **Meth-1** computed using the PBE functional in the gas phase.

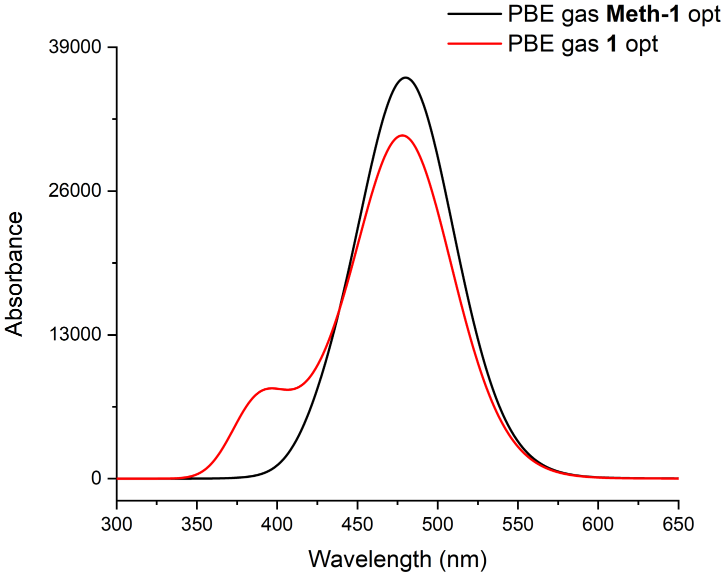

Figure S16: Comparison between the absorption spectra obtained with the PBE functional for the molecules **1** and **Meth-1** in the gas phase.

S3.4 PBE results in solution

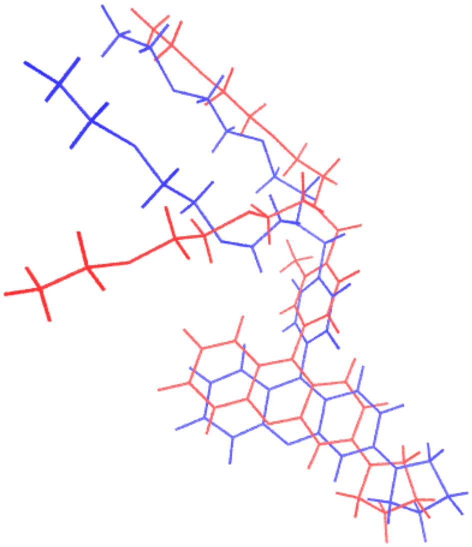

Figure S17: Comparison between the optimized structures of the **1** (blue) and **Meth-1** (red) molecules calculated using the PBE functional in solution.

All consideration drawn in the case of PBE in the gas phase hold.

Table S6: Excitation energy (E, in eV, and λ in nm), oscillator strength (f) and molecular orbitals involved in each electronic transition computed at PBE level for the molecules 1 and Meth-1 in solution.

		**1**	**Meth-1**
Excited state 1	E (eV)	1.9200	1.9300
	λ (nm)	645.75	642.40
	f	0.0006	0.0001
	MOs (c)	177 → 179 (0.70683)	181 → 183 (0.70702)
Excited state 2	E (eV)	2.0631	2.0312
	λ (nm)	600.96	610.41
	f	0.0006	0.0012
	MOs (c)	176 → 179 (0.70689)	180 → 183 (0.70575)
Excited state 3	E (eV)	2.0795	2.0751
	λ (nm)	596.21	597.49
	f	0.0005	0.0012
	MOs (c)	175 → 179 (0.70684)	179 → 183 (0.70613)
Excited state 4	E (eV)	2.2160	2.2244
	λ (nm)	559.49	557.39
	f	0.9954	1.0033
	MOs (c)	178 → 179 (0.70176)	182 → 183 (0.69907)
Excited state 5	E (eV)	2.2619	2.2667
	λ (nm)	548.13	546.97
	f	0.0007	0.0008
	MOs (c)	173 → 179 (-0.12930)	177 → 183 (0.32829)
		174 → 179 (0.69486)	178 → 183 (0.62492)

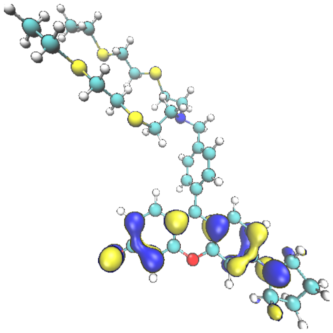

(a) ***1*** *molecular orbital 178 (HOMO).*

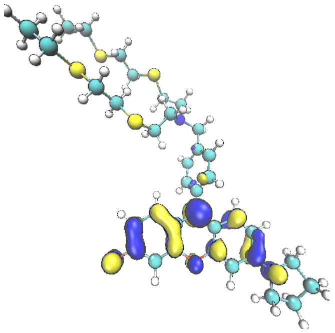

(b) ***1*** *molecular orbital 179 (LUMO).*

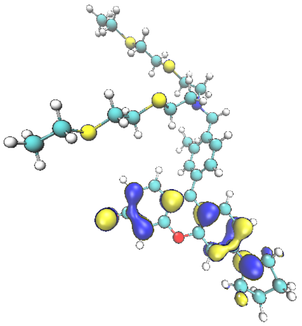

(c) ***Meth-1*** *molecular orbital 182 (HOMO).*

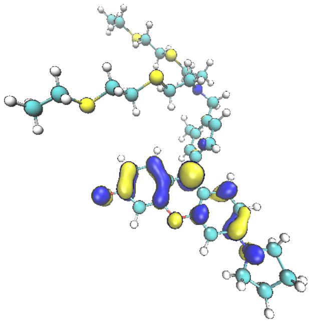

(d) ***Meth-1*** *molecular orbital 183 (LUMO).*

Figure S18: Most relevant orbitals of the molecules **1** and **Meth-1** computed using the PBE functional in solution.

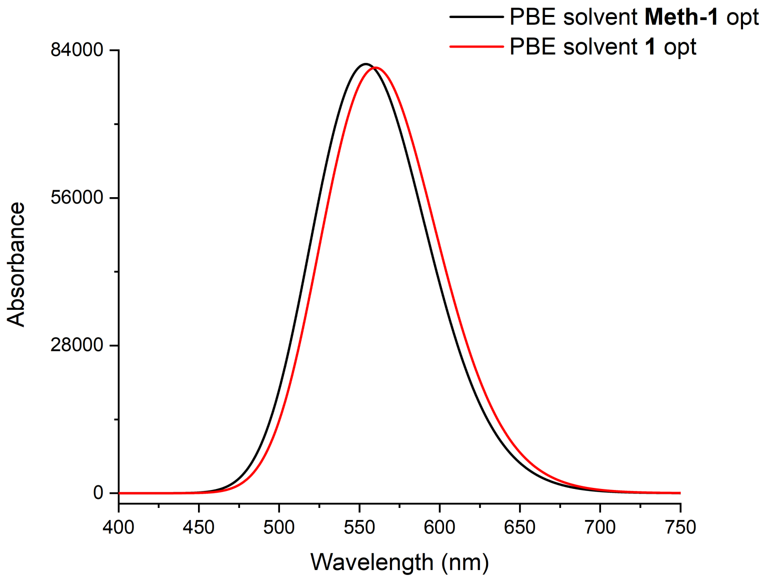

Figure S19: Comparison between the absorption spectra obtained with the PBE functional for the molecules **1** and **Meth-1** in solution.

S3.5 B3LYP results in the gas phase

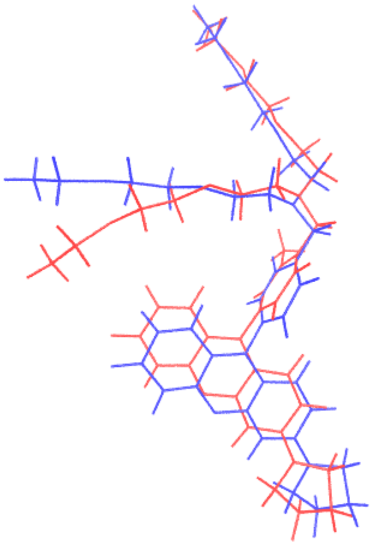

Figure S20: Comparison between the optimized structures of the **1** (blue) and **Meth-1** (red) molecules calculated using the B3LYP functional in the gas phase.

Table S7: Excitation energy (E, in eV, and λ in nm), oscillator strength (f) and molecular orbitals involved in each electronic transition computed at B3LYP level for the molecules 1 and Meth-1 in the gas phase.

		**1**	**Meth-1**
Excited state 1	E (eV)	2.7613	2.7689
	λ (nm)	449.01	447.78
	f	0.0001	0.0011
	MOs (c)	175 → 179 (0.69531)	178 → 183 (0.57222)
			179 → 183 (0.15395)
			180 → 183 (0.20301)
			181 → 183 (-0.30529)
Excited state 2	E (eV)	2.8719	2.8798
	λ (nm)	431.72	430.53
	f	0.5759	0.6164
	MOs (c)	170 → 179 (-0.10209)	182 → 183 (0.68381)
		178 → 179 (0.68474)	
Excited state 3	E (eV)	3.1314	2.9526
	λ (nm)	395.94	419.92
	f	0.0001	0.0038
	MOs (c)	177 → 179 (0.70147)	178 → 183 (0.28601)
			181 → 183 (0.63559)
Excited state 4	E (eV)	3.3326	3.2319
	λ (nm)	372.03	383.62
	f	0.1038	0.0005
	MOs (c)	170 → 179 (-0.11466)	178 → 183 (-0.23765)
		172 → 179 (0.12035)	180 → 183 (0.65792)
		173 → 179 (0.66648)	
		178 → 179 (-0.10549)	
Excited state 5	E (eV)	3.3901	3.3418
	λ (nm)	365.72	371.01
	f	0.0004	0.1033
	MOs (c)	174 → 179 (0.45268)	174 → 183 (0.11281)
		176 → 179 (0.53470)	177 → 183 (0.67563)
			182 → 183 (-0.10259)

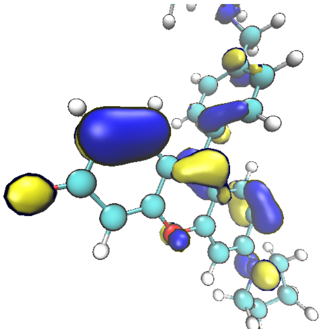

(a) ***1*** *molecular orbital 170.*

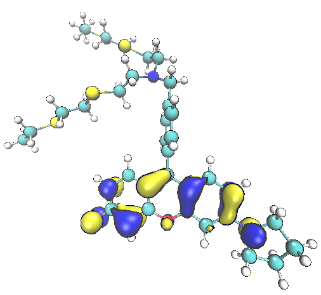

(b) ***1*** *molecular orbital 178 (HOMO).*

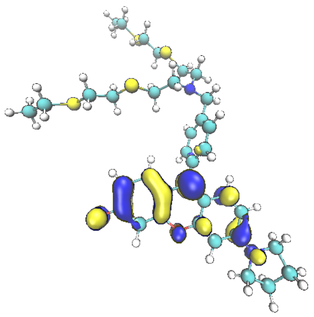

(c) ***1*** *molecular orbital 179 (LUMO).*

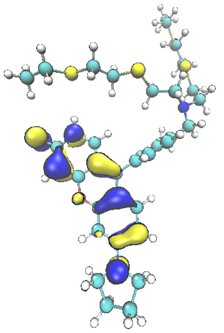

(d) ***Meth-1*** *molecular orbital 182 (HOMO).*

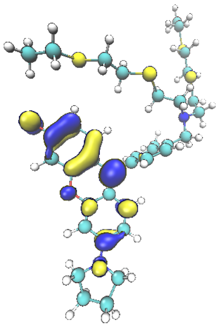

(e) ***Meth-1*** *molecular orbital 183 (LUMO).*

Figure S21: Most relevant orbitals of the molecules **1** and **Meth-1** computed using the B3LYP functional in the gas phase.

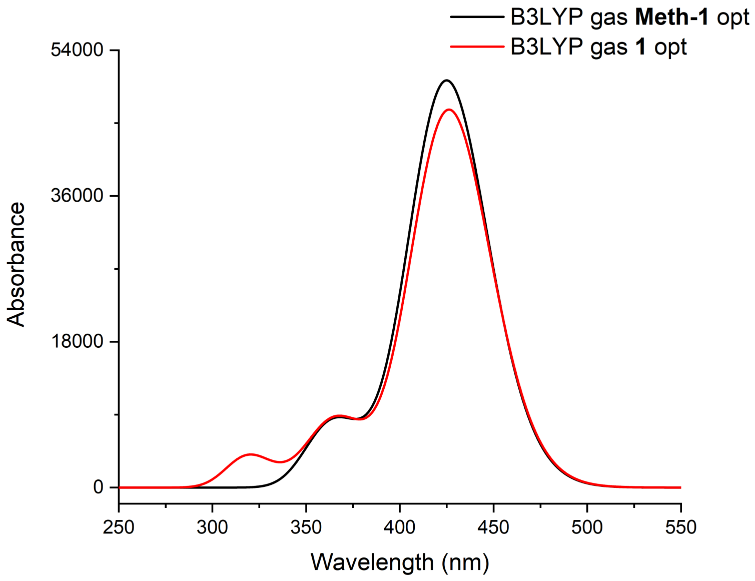

Figure S22: Comparison between the absorption spectra obtained with the B3LYP functional for the **1** and **Meth-1** molecules in the gas phase.

S3.6 B3LYP results in solution

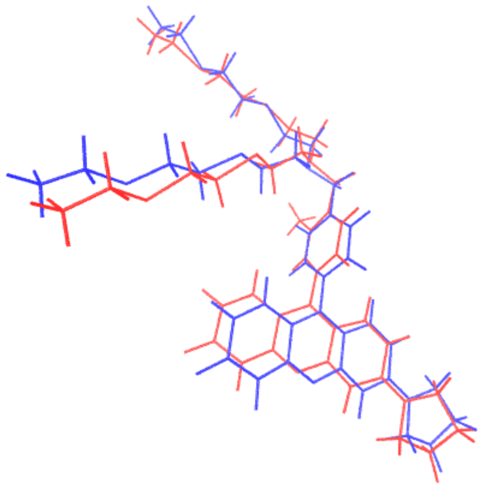

Figure S23: Comparison between the optimized structures of the **1** (blue) and **Meth-1** (red) molecules calculated using the B3LYP functional in solution.

Table S8: Excitation energy (E, in eV, and λ in nm), oscillator strength (f) and molecular orbitals involved in each electronic transition computed at B3LYP level for the molecules 1 and Meth-1 in solution.

		**1**	**Meth-1**
Excited state 1	E (eV)	2.4780	2.4765
	λ (nm)	500.35	500.63
	f	1.2122	1.2036
	MOs (c)	178 → 179 (0.70455)	182 → 183 (0.70455)
Excited state 2	E (eV)	3.1408	3.1323
	λ (nm)	394.76	395.83
	f	0.0320	0.0070
	MOs (c)	174 → 179 (0.22024)	180 → 183 (0.20187)
		175 → 179 (-0.30797)	181 → 183 (0.65698)
		177 → 179 (0.57859)	
Excited state 3	E (eV)	3.1827	3.1739
	λ (nm)	389.55	390.64
	f	0.0106	0.0305
	MOs (c)	170 → 179 (-0.12604)	178 → 183 (0.67023)
		174 → 179 (0.64741)	180 → 183 (0.10424)
		177 → 179 (-0.21239)	
Excited state 4	E (eV)	3.2087	3.1949
	λ (nm)	386.40	388.06
	f	0.0007	0.0001
	MOs (c)	171 → 179 (0.35914)	175 → 183 (0.13670)
		176 → 179 (0.59958)	179 → 183 (0.40322)
			180 → 183 (0.54426)
			181 → 183 (-0.10555)
Excited state 5	E (eV)	3.2114	3.2103
	λ (nm)	386.07	386.21
	f	0.0002	0.0013
	MOs (c)	171 → 179 (0.58358)	174 → 183 (0.12122)
		176 → 179 (-0.35027)	175 → 183 (0.66852)
		177 → 179 (-0.12465)	

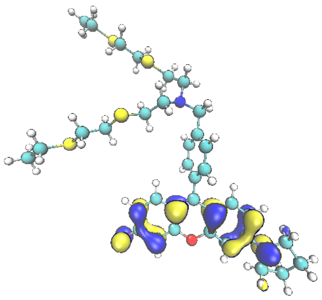

(a) ***1*** *molecular orbital 178 (HOMO).*

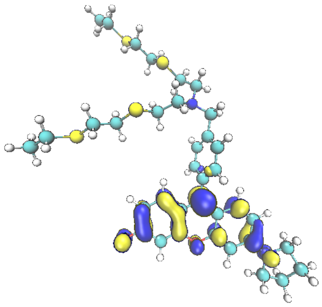

(b) ***1*** *molecular orbital 179 (LUMO).*

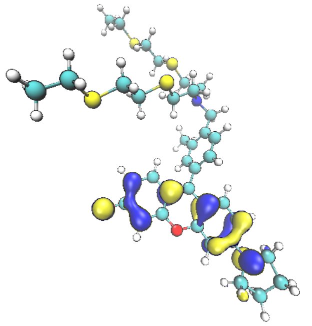

(c) ***Meth-1*** *molecular orbital 182 (HOMO).*

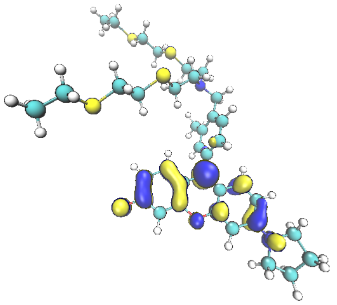

(d) ***Meth-1*** *molecular orbital 183 (LUMO).*

Figure S24: Most relevant orbitals of the molecules **1** and **Meth-1** computed using the B3LYP functional in solution.

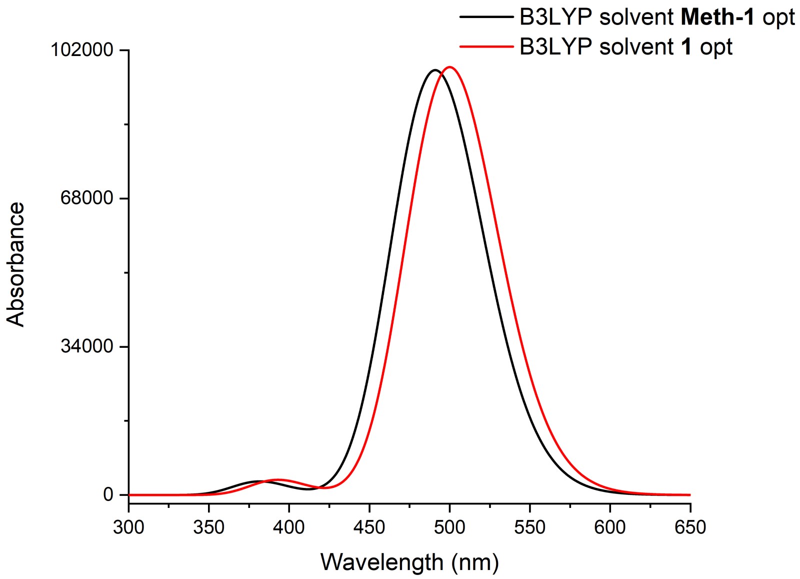

Figure S25: Comparison between the absorption spectra obtained with the B3LYP functional for the molecules **1** and **Meth-1** in solution.

S3.7 BLYP results in the gas phase

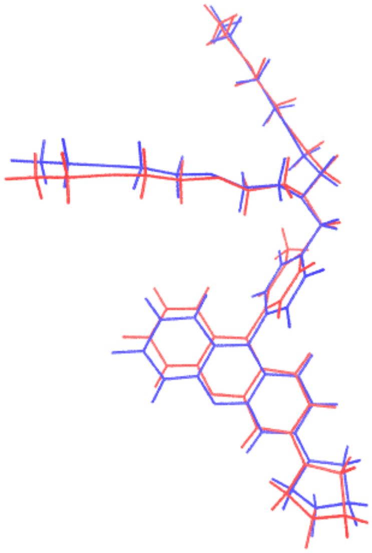

Figure S26: Comparison between the optimized structures of the **1** (blue) and **Meth-1** (red) molecules calculated using the BLYP functional in the gas phase.

Table S9: Excitation energy (E, in eV, and λ in nm), oscillator strength (f) and molecular orbitals involved in each electronic transition computed at BLYP level for the molecules 1 and Meth-1 in the gas phase.

		**1**	**Meth-1**
Excited state 1	E (eV)	1.9074	1.9047
	λ (nm)	650.02	650.95
	f	0.0000	0.0001
	MOs (c)	177 → 179 (0.70591)	181 → 183 (0.70530)
Excited state 2	E (eV)	1.9611	1.9665
	λ (nm)	632.21	630.48
	f	0.0000	0.0000
	MOs (c)	176 → 179 (0.70695)	180 → 183 (0.70667)
Excited state 3	E (eV)	2.1772	2.1826
	λ (nm)	569.45	568.05
	f	0.0001	0.0001
	MOs (c)	175 → 179 (0.70698)	179 → 183 (0.70705)
Excited state 4	E (eV)	2.2204	2.2360
	λ (nm)	558.38	554.50
	f	0.0001	0.0000
	MOs (c)	174 → 179 (0.70710)	178 → 183 (0.70709)
Excited state 5	E (eV)	2.3291	2.3349
	λ (nm)	532.33	531.00
	f	0.0013	0.0001
	MOs (c)	173 → 179 (0.70616)	177 → 183 (0.70695)
Excited state 6	E (eV)	2.5011	2.4984
	λ (nm)	495.72	496.26
	f	0.0023	0.0001
	MOs (c)	171 → 179 (0.23916)	176 → 183 (0.70506)
		172 → 179 (0.66282)	
Excited state 7	E (eV)	2.5293	2.5260
	λ (nm)	490.20	490.84
	f	0.3912	0.3895
	MOs (c)	170 → 179 (0.13766)	174 → 183 (0.12975)
		171 → 179 (0.13967)	175 → 183 (0.12444)
		178 → 179 (0.65941)	182 → 183 (0.66390)
		178 → 180 (0.10799)	182 ← 183 (-0.11074)
		178 ← 179 (-0.11027)	
Excited state 8	E (eV)	2.7563	2.7551
	λ (nm)	449.83	450.02
	f	0.1080	0.1083

	MOs (c)	171 → 179 (0.63075)	175 → 183 (0.67309)
		172 → 179 (-0.23806)	182 → 183 (-0.12036)
		178 → 179 (-0.12181)	182 → 184 (-0.10195)
Excited state 9	E (eV)	3.0335	3.0884
	λ (nm)	408.71	401.45
	f	0.0007	0.0005
	MOs (c)	177 → 180 (0.68880)	181 → 184 (0.69548)
		178 → 180 (-0.15677)	182 → 184 (-0.12228)
Excited state 10	E (eV)	3.0601	3.1164
	λ (nm)	405.17	397.85
	f	0.0550	0.0602
	MOs (c)	177 → 180 (0.15332)	181 → 184 (0.11821)
		178 → 179 (-0.10169)	182 → 184 (0.65315)
		178 → 180 (0.65477)	182 → 185 (-0.17936)
		178 → 181 (-0.10662)	
		178 → 182 (0.11242)	

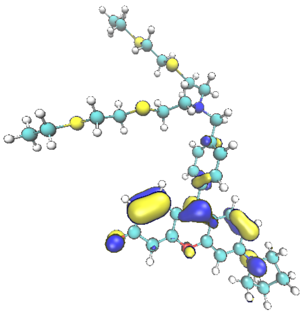

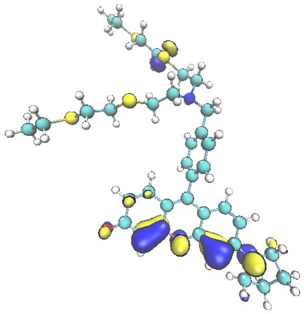

(a) ***1*** *molecular orbital 170.*

(b) ***1*** *molecular orbital 171.*

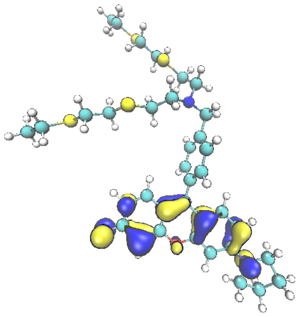

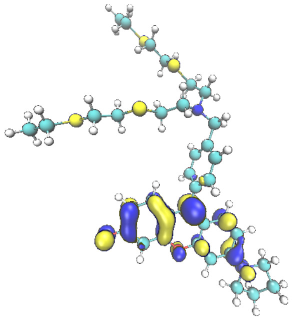

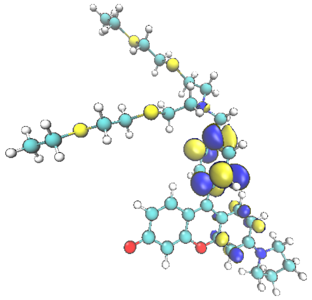

(c) ***1*** *molecular orbital 178 (HOMO).*

(d) ***1*** *molecular orbital 179 (LUMO).*

(e) ***1*** *molecular orbital 180.*

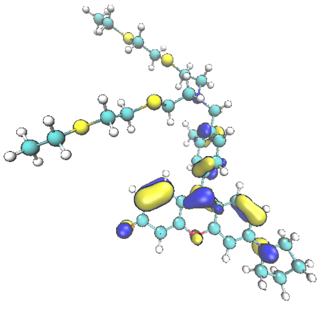

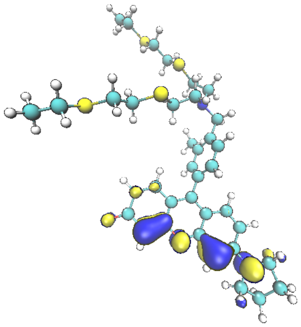

(f) ***Meth-1*** *molecular orbital 174.*

(g) ***Meth-1*** *molecular orbital 175.*

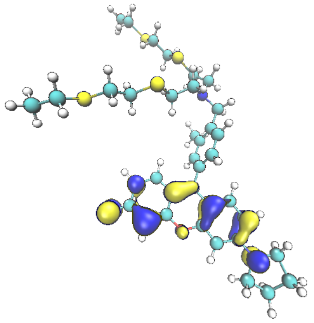

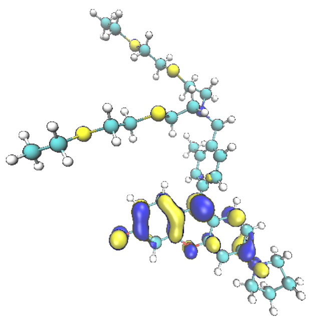

(h) ***Meth-1*** *molecular orbital 182 (HOMO).*

(i) ***Meth-1*** *molecular orbital 183 (LUMO).*

Figure S27: Most relevant orbitals of the molecules **1** and **Meth-1** computed using the BLYP functional in the gas phase.

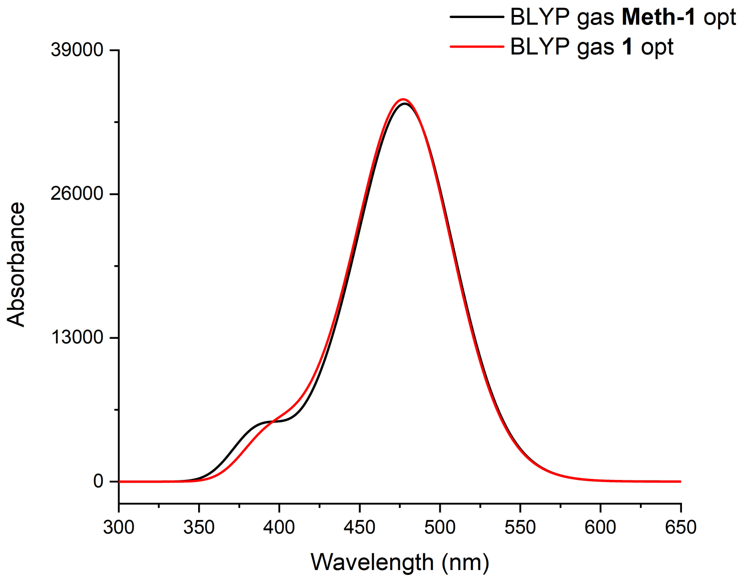

Figure S28: Comparison between the absorption spectra obtained with the BLYP functional for the molecules **1** and **Meth-1** in the gas phase.

S3.8 BLYP results in solution

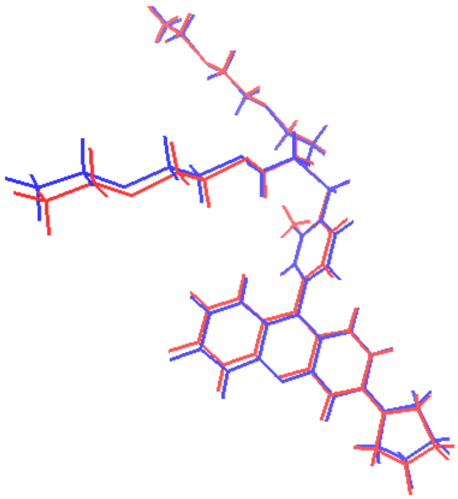

Figure S29: Comparison between the optimized structures of the **1** (blue) and **Meth-1** (red) molecules calculated using the BLYP functional in solution.

Table S10: Excitation energy (E, in eV, and λ in nm), oscillator strength (f) and molecular orbitals involved in each electronic transition computed at BLYP level for the molecules 1 and Meth-1 in solution.

		**1**	**Meth-1**
Excited state 1	E (eV)	1.9946	1.9800
	λ (nm)	621.61	626.18
	f	0.0024	0.0001
	MOs (c)	177 → 179 (0.70657)	181 → 183 (0.70705)
Excited state 2	E (eV)	2.0781	2.0841
	λ (nm)	596.61	594.92
	f	0.0001	0.0004
	MOs (c)	176 → 179 (0.70705)	180 → 183 (0.70676)
Excited state 3	E (eV)	2.1021	2.1117
	λ (nm)	589.82	587.14
	f	0.0003	0.0002
	MOs (c)	175 → 179 (0.70694)	179 → 183 (0.70695)
Excited state 4	E (eV)	2.2092	2.2105
	λ (nm)	561.21	560.89
	f	1.0155	1.0124
	MOs (c)	178 → 179 (0.70200)	182 → 183 (0.70214)
Excited state 5	E (eV)	2.3018	2.3116
	λ (nm)	538.63	536.37
	f	0.0001	0.0004
	MOs (c)	173 → 179 (0.70055)	177 → 183 (0.70624)

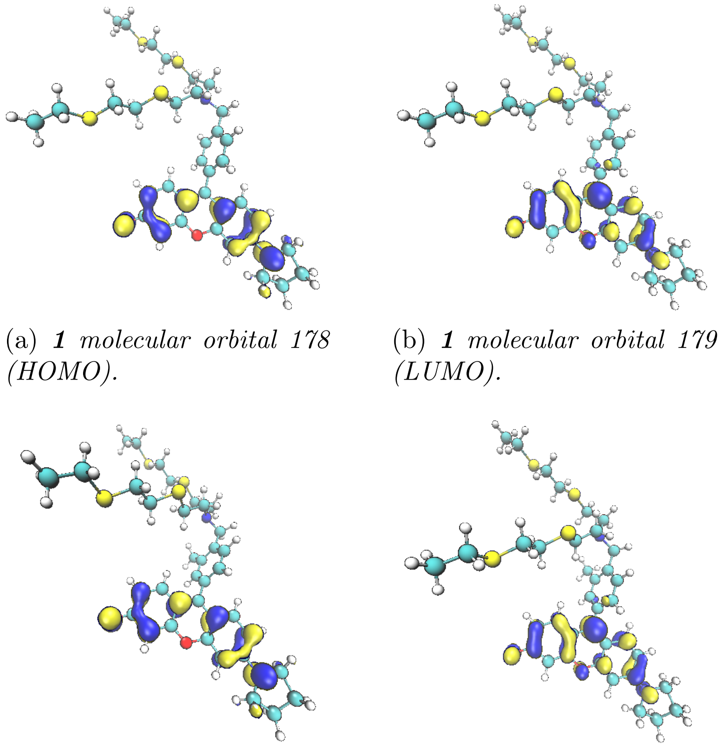

(a) ***1** molecular orbital 178 (HOMO).*

(b) ***1** molecular orbital 179 (LUMO).*

(c) ***Meth-1** molecular orbital 182 (HOMO).*

(d) ***Meth-1** molecular orbital 183 (LUMO).*

Figure S30: Most relevant orbitals of the molecules **1** and **Meth-1** computed using the BLYP functional in solution.

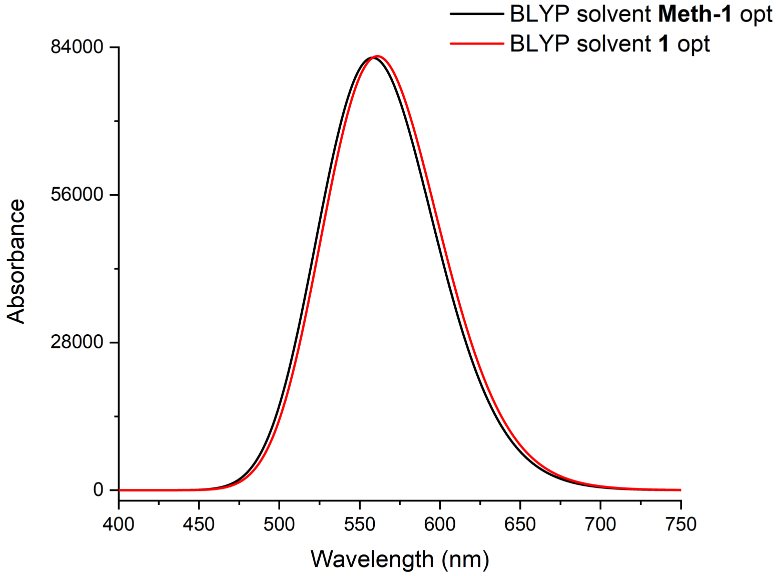

Figure S31: Comparison between the absorption spectra obtained with the BLYP functional for the molecules **1** and **Meth-1** in solution.
